# Impact of a pay-for-performance scheme for long-acting reversible contraceptive (LARC) advice on contraceptive uptake and abortion in British primary care: An interrupted time series study

**DOI:** 10.1371/journal.pmed.1003333

**Published:** 2020-09-14

**Authors:** Richard Ma, Elizabeth Cecil, Alex Bottle, Rebecca French, Sonia Saxena

**Affiliations:** 1 Department of Primary Care and Public Health, Imperial College London, London, United Kingdom; 2 Department of Public Health, Environments and Society, London School of Hygiene and Tropical Medicine, London, United Kingdom; Harvard Medical School, UNITED STATES

## Abstract

**Background:**

Long-acting reversible contraception (LARC) is among the most effective contraceptive methods, but uptake remains low even in high-income settings. In 2009/2010, a target-based pay-for-performance (P4P) scheme in Britain was introduced for primary care physicians (PCPs) to offer advice about LARC methods to a specified proportion of women attending for contraceptive care to improve contraceptive choice. We examined the impact and equity of this scheme on LARC uptake and abortions.

**Methods and findings:**

We examined records of 3,281,667 women aged 13 to 54 years registered with a primary care clinic in Britain (England, Wales, and Scotland) using Clinical Practice Research Datalink (CPRD) from 2004/2005 to 2013/2014. We used interrupted time series (ITS) analysis to examine trends in annual LARC and non-LARC hormonal contraception (NLHC) uptake and abortion rates, stratified by age and deprivation groups, before and after the P4P was introduced in 2009/2010. Between 2004/2005 and 2013/2014, crude LARC uptake rates increased by 32.0% from 29.6 per 1,000 women to 39.0 per 1,000 women, compared with 18.0% decrease in NLHC uptake. LARC uptake among women of all ages increased immediately after the P4P with step change of 5.36 per 1,000 women (all values are per 1,000 women unless stated, 95% CI 5.26–5.45, *p <* 0.001). Women aged 20 to 24 years had the largest step change (8.40, 8.34–8.47, *p <* 0.001) and sustained trend increase (3.14, 3.08–3.19, *p <* 0.001) compared with other age groups. NLHC uptake fell in all women with a step change of −22.8 (−24.5 to −21.2, *p <* 0.001), largely due to fall in combined hormonal contraception (CHC; −15.0, −15.5 to −14.5, *p <* 0.001). Abortion rates in all women fell immediately after the P4P with a step change of −2.28 (−2.98 to −1.57, *p =* 0.002) and sustained decrease in trend of −0.88 (−1.12 to −0.63, *p <* 0.001). The largest falls occurred in women aged 13 to 19 years (step change −5.04, −7.56 to −2.51, *p =* 0.011), women aged 20 to 24 years (step change −4.52, −7.48 to −1.57, *p =* 0.030), and women from the most deprived group (step change −4.40, −6.89 to −1.91, *p =* 0.018). We estimate that by 2013/2014, the P4P scheme resulted in an additional 4.53 LARC prescriptions per 1,000 women (relative increase of 13.4%) more than would have been expected without the scheme. There was a concurrent absolute reduction of −5.31 abortions per 1,000 women, or −38.3% relative reduction. Despite universal coverage of healthcare, some women might have obtained contraception elsewhere or had abortion procedure that was not recorded on CPRD. Other policies aiming to increase LARC use or reduce unplanned pregnancies around the same time could also explain the findings.

**Conclusions:**

In this study, we found that LARC uptake increased and abortions fell in the period after the P4P scheme in British primary care, with additional impact for young women aged 20–24 years and those from deprived backgrounds.

## Abstract 2

Richard Ma and colleagues study a scheme to improve uptake of long-acting reversible contraception in primary care in England, Scotland, and Wales.

## Author summary

**Why was this study done?:**

- Long-acting reversible contraception (LARC) is more effective and longer lasting than other methods such as condoms or contraceptive pills at controlling fertility.
- Primary care physicians (PCPs) in Britain were given financial incentives to give information on LARC methods to over 90% of women coming for contraceptive advice from 2009/2010.
- We wanted to find out whether this new scheme led to more LARC prescribing and fewer abortions, signifying fewer unplanned and unwanted pregnancies, particularly in younger women and those from poorer backgrounds.

**What did the researchers do and find?:**

- We studied health records of over 3 million women aged 13 to 54 years old, from over 600 primary care practices across Britain (England, Wales, and Scotland) from financial years 2004/2005 to 2013/2014.
- We compared yearly LARC prescriptions and abortions in the years before and after the LARC advice scheme was introduced in 2009/2010.
- We found 13% more LARC prescriptions and 38% fewer abortions than expected since the scheme was introduced, particularly in younger women aged 20–24 and those from poorer backgrounds.

**What do these findings mean?:**

- Financial incentives for PCPs to give LARC advice to women were associated with more LARC prescriptions and reductions in abortions.
- The greater impact in younger women and those from more disadvantaged backgrounds suggests potential to reduce health inequalities by improved awareness of options for effective fertility control.

## Introduction

Unintended pregnancies make up an estimated 44% of all pregnancies worldwide, nearly 60% of these end in abortions [1], and up to half of unintended pregnancies are due to incorrect or inconsistent use of contraception [2,3]. Britain has among the highest teenage abortion rates in Europe [4,5]; together, these suggest significant room for improvement in contraceptive programmes in Britain and worldwide [6].

Long-acting reversible contraception (LARC) methods include contraceptive injections, subdermal implants, intrauterine devices (IUDs), and the intrauterine systems (IUSs); they offer continuous contraception from 3 months (injection) to 10 years (IUD). LARCs are reliable, safe, and more effective at preventing unintended pregnancy compared with combined hormonal contraception (CHC) and progestogen-only pills (POP). Therefore, their use is recommended by global programmes and national guidance [7,8]; despite these features, the uptake of LARC is lower than 15% in many countries [9,10]. Although LARC methods are long-lasting, those using injection must remember to have it every 12 weeks for continuous contraception.

In Britain, women have access to free contraception, and primary care is the most commonly used and preferred setting to obtain contraception [11,12]. In 2004/2005, the British National Health Service (NHS) introduced radical reforms in primary care through a pay-for-performance (P4P) scheme that linked primary care practices’ income to performance targets. A new set of targets were introduced in 2009/2010 that remunerated primary care physicians (PCPs) to offer full choice of contraception including advice about LARC to women aged 13 to 54 years attending for contraceptive care [13]. Under the scheme, PCPs could offer advice in verbal or written format and could do so in person, via text message, or letter. Primary care practices were paid for achieving targets for advice only but not LARC uptake.

The number of LARC items prescribed increased following this scheme, but its impact on intended outcomes such as LARC uptake and abortion in the population have not been reported [14]. Our main objective was to examine the impact of a P4P scheme for LARC advice on LARC uptake and abortion rates in women attending British primary care. Our secondary objective was to assess the impact of the scheme on LARC uptake and abortions among young women aged less than 25 years and those from deprived backgrounds, who have relatively higher abortion rates. We hypothesised that LARC uptake would increase after the P4P scheme, and that this would lead to reduction in abortions.

## Methods

### Data source

We used Clinical Practice Research Datalink (CPRD), a database of anonymised electronic health records extracted from over 600 primary care practices in Britain (i.e. England, Scotland, and Wales). It has a coverage of over 17 million registered patients broadly representative of age, sex, and ethnicity of approximately 7% of the British population [15].

### Population

We extracted records for women aged 13 to 54 years, which was the target age range for the P4P scheme. For each financial year (1 April to 31 March the following year) from 2004/2005 to 2013/2014, we included women in the denominator if they were registered with a primary care practice at any point that year that was deemed “up to standard” (when the practice met data quality and completeness criteria) in the previous year. The population was an open cohort, and new women were added to the denominator population each year. Women became eligible when they turned 13 in any study year; conversely, women who turned 55 were censored, as were any who died, or if their practice stopped contributing data to CPRD.

We assigned women to one of 4 age groups: 13 to 19, 20 to 24, 25 to 34, and 35 to 54 years; women could pass across age groups over the study period. Women were assigned to one of 5 population-weighted deprivation groups based on their residential post code using English Indices of Multiple Deprivation (IMD) quintiles that range from IMD 1 (least deprived) to IMD 5 (most deprived). The IMD is a weighted combination measure of deprivation using 38 separate indicators across 7 distinct domains identified in the English Indices of Deprivation: deprivation, employment deprivation, health deprivation and disability, education skills and training deprivation, barriers to housing and services, living environment deprivation, and crime; IMD data are available for English populations only [16]. The postcode used to derive deprivation groups is unique for women in the cohort during the time they were registered to the practice. When the patient moved and registered with a new practice, that individual was censored from the previous practice.

Every woman between the ages of 13 and 54 registered with a practice for any part of the financial year was potentially “at risk” of unplanned pregnancy. We used this definition as it was consistent with the denominator defined as eligible for LARC advice in the P4P scheme.

### Outcomes

Our main outcome was annual LARC uptake, calculated by summing the total number of women who chose any LARC method divided by the number of women registered in the denominator that financial year, expressed as per 1,000 women aged 13–54 years. We defined LARC as any branded or generic prescriptions of contraceptive injections, implants, IUSs, and IUDs. All other branded or generic prescriptions of CHCs (including pills, vaginal rings, and patches) and POPs were grouped as non-LARC hormonal contraception (NLHC). We reported both NLHC and LARC prescriptions to examine overall contraceptive uptake and changes in method preference over the years.

Given that our main aim was to evaluate impact of a P4P on LARC uptake, we only recorded whether LARC was prescribed at all for each woman in any given year, rather than quantifying the number of such prescriptions. LARC methods such as implants and IUDs last from 3 to 10 years and so were prescribed infrequently, whereas injections and NLHC were prescribed several times a year. Therefore, for fairer comparison, we counted only the incident prescription of either a LARC or NLHC per woman per year, whichever was prescribed first. If women were prescribed more than one LARC product, we counted only the first product that year.

Our secondary outcome measure was annual abortion rates, defined as the number of abortions divided by denominator count, expressed as per 1,000 women aged 13–54 years. We used abortion as a proxy measure of unplanned and unwanted pregnancy; notwithstanding the limitations, we hypothesised this would be reduced if more women chose to use more effective LARC methods.

Women in Britain are referred for an abortion through their PCP, hospital, or sexual health clinic; they can also self-refer in some areas. Women may also withhold consent for information to be sent back to their PCP; for this reason and to minimise missing data, we compiled a list that included both referral and procedure codes for abortions.

Our analysis focused on Britain (i.e., England, Scotland, and Wales) rather than the whole of the United Kingdom as access to abortion was restricted in Northern Ireland during the study period. Women often travelled to Britain to have an abortion privately, which renders Northern Ireland’s abortion figures unreliable. Women may have more than 1 abortion in any year, but we counted only the earliest event if there were 2 or more recorded within 6 weeks of another to be biologically plausible.

We calculated annual rates for LARC, NLHC, and abortion and performed subgroup analyses by age and deprivation.

### Statistical analysis

We examined outcomes using interrupted time series (ITS) analysis to compare trends in annual contraceptive uptake and abortion rates before and after the introduction of the P4P in financial year 2009/2010. ITS analysis is a strong quasi-experimental research design often used to evaluate impact of natural experiments such as health policy changes at a population level [17].

We used a segmented time series regression model for analysis. The regression is represented by S1 Fig and the formula *outcome_jt_* = *β*_0_+*β*_1_∙*time_t_*+*β*_2_∙*level_j_*+*β*_3_∙*trend_jt_*+*ε_jt_*. Any outcome of intervention status *j* at time *t* is a product of 4 variables: the existing level *β*_0_ at time *0;* *β*_1_ represents the trend at time *t;* *β*_2_ is the step change in level for intervention *j*; *β*_3_ represents the trend line at time *t* for intervention *j*; with additional error terms for that time and intervention.

To model possible long-term seasonal patterns, the data were “time stratified” by year, which permitted adjustment for confounding by “seasonality.” We used 5 time points for the pre-intervention phase from 2004/2005 to 2008/2009; we excluded data for 2009/2010 in the analysis to allow for a 1-year “phase-in period” for the scheme to take effect, followed by 4 time points after the phase-in period from 2010/2011 to 2013/2014. To account for auto-correlated data, we used autoregressive moving average (ARMA) models [18]. The order of the moving average and the autoregressive model parameters were determined using multiple methods, including scatter plots of the deviance residuals versus time, the Durbin–Watson test, and the autocorrelation and partial autocorrelation functions [17,19,20]. We used the maximum likelihood ratio test to assess the fit of the model parameters. We used the Generalised Least Squares (GLS) approach to run the final model, which is like a linear regression but allowed autoregressive and moving average processes.

We used the final model to estimate the absolute and relative changes in 2013/2014, i.e., 4 years after the P4P scheme was introduced, by calculating the differences between the counterfactual (which is an extension of the pre-P4P trendline) and fitted model (post-P4P trendline) at that time point. There are no precision parameters for these because they are derived from the modelled trends, which already have their confidence intervals.

### Sample size

Population studies suggest that the lowest prevalence of LARC use was 1 per 1,000 for intrauterine device (IUD) in women of reproductive ages [9,10]; an increase of 15% is both clinically significant and likely, given that previous studies reported a range of 6%–20% [14]. A sample size of 1,016,000 women before and after intervention would be adequate to detect an increase in proportion from 1 in 1,000 to 1.15 in 1,000 with 90% power at 5% significance level.

### Sensitivity analysis

Although the incentive was introduced in financial year 2009/2010, we allowed one full financial year for it to be fully implemented (“phase-in period”); this gave women time to respond to advice and start or switch to LARC. We compared the trends in crude contraceptive prescription and abortion rates with (5 time points before, 1 year of “phase-in,” and 4 time points after) and without phase-in period (5 time points before the intervention and 5 time points after).To demonstrate the time taken to fully implement the P4P scheme, we calculated the proportion of eligible women based on the P4P scheme (those prescribed NLHC) given LARC advice per financial year from 2004/2005 to 2013/2014.

Women have been able to self-refer for abortions without having to see their PCP. They also could withhold consent for the clinic to inform their PCP of the abortion procedure. To examine the impact of missing abortion events, we conducted a sensitivity analysis to examine the effect of adding 20%, 40%, and 50% extra abortion events after the P4P scheme.

We used STATA version 14 (STATA Corp, College Station, TX) to perform the data management. We used R Studio (Version 1.1.456–2009–2018 RStudio; http://www.r-project.org) for modelling and statistical tests.

### Patient and public involvement

This research has involved views of the public throughout; this included informal engagement on social media at the time of RM’s doctoral fellowship proposal, and then more formally through a Project Advisory Group (PAG) that meets twice a year to discuss the programme of research during the fellowship. Membership of PAG includes individuals, professionals, and charity representatives in the sexual and reproductive healthcare sector. Members’ contributions have included lay summaries, helping with sensitive use of language, framing, interpretation, reporting, and dissemination of study findings.

### Ethical approval

The CPRD has obtained ethical approval from the UK’s National Research Ethics Service (NRES) Committee for observational research using anonymised data. The study protocol was approved by the Independent Scientific Advisory Committee (ISAC) for Medicines and Healthcare Products Regulatory Agency (MHRA) database research (protocol number 15/076_R2, S1 File).

## Results

Our study population included 3,281,667 women aged 13 to 54 years from over 600 practices across Britain from 2004/2005 to 2013/2014. The total eligible population, and the relative proportions by age group, region, and deprivation groups for each financial year, were broadly stable across the study period (S2–S4 Figs). There was a 12.9% attrition in the annual denominator population from 1,117,648 in 2004/2005 to 973,473 in 2013/2014 (Table 1) due to reduced number of practices contributing to CPRD as several practices switched software systems in a few of regions in England (North East, Yorkshire and Humber and East Midlands). Practice populations in the South of England and more affluent postcodes were overrepresented. LARC advice was offered to 43.4% of eligible women in 2004/2005, rising to 78.3% in 2008/2009 and then 95.3% when the P4P was introduced in 2009/2010; this proportion was sustained between 96.5% and 97.9% by the end of the study period, which supported the use of a phase-in period in 2009/2010 (S1A Table).

**Table 1:** Crude rates per 1,000 women for LARC, NLHC uptake, and abortion by age and deprivation groups at 3 time points: Study baseline (2004/2005), introduction of P4P scheme in 2009/2010, and end of study (2013/2014).

Variable	LARC			NLHC			Abortions		
Year	2004/2005	2009/2010	2013/2014	2004/2005	2009/2010	2013/2014	2004/2005	2009/2010	2013/2014
**All ages**	*1*,*117*,*648*	*1*,*156*,*125*	*973*,*473*	*1*,*117*,*648*	*1*,*156*,*125*	*973*,*473*	*1*,*117*,*648*	*1*,*156*,*125*	*973*,*473*
	**29.6**	**33.9**	**39.0**	**172**	**208**	**203**	**16.9**	**13.9**	**8.59**
**Age group (years)**									
13–19	159,368	163,444	139,621	150,646	163,444	139,621	150,646	163,444	139,621
	**20.9**	**24.0**	**27.6**	**197**	**223**	**205**	**26.6**	**18.1**	**7.66**
20–24	107,921	113,168	97,039	103,018	113,168	97,039	103,018	113,168	97,039
	**52.9**	**52.4**	**63.0**	**393**	**434**	**396**	**57.5**	**44.4**	**23.8**
25–34	252,564	253,811	222,146	253,424	253,811	222,146	253,424	253,811	222,146
	**45.7**	**47.8**	**54.2**	**291**	**343**	**323**	**25.2**	**22.7**	**15.8**
35–54	629,744	625,702	514,667	610,560	625,702	514,667	610,560	625,702	514,667
	**21.0**	**27.4**	**31.1**	**79.1**	**108**	**114**	**4.17**	**3.63**	**2.85**
Deprivation* **group**									
Least deprived	161,781	177,608	141,254	161,781	177,608	141,254	161,781	177,608	141,254
	**22.0**	**25.9**	**31.6**	**166**	**210**	**207**	**12.1**	**9.12**	**4.96**
Most deprived	106,615	120,866	98,300	106,615	120,866	98,300	106,615	120,866	98,300
	**42.4**	**44.4**	**48.3**	**161**	**192**	**172**	**26.5**	**20.6**	**12.9**

*Deprivation data available for England only.

Denominators are given in italics and rate per 1,000 women in bold.

**Abbreviations:** LARC, long-acting reversible contraception; NLHC, non-LARC hormonal contraception; P4P, pay for performance

### Trends in LARC uptake

**Fig 1:**
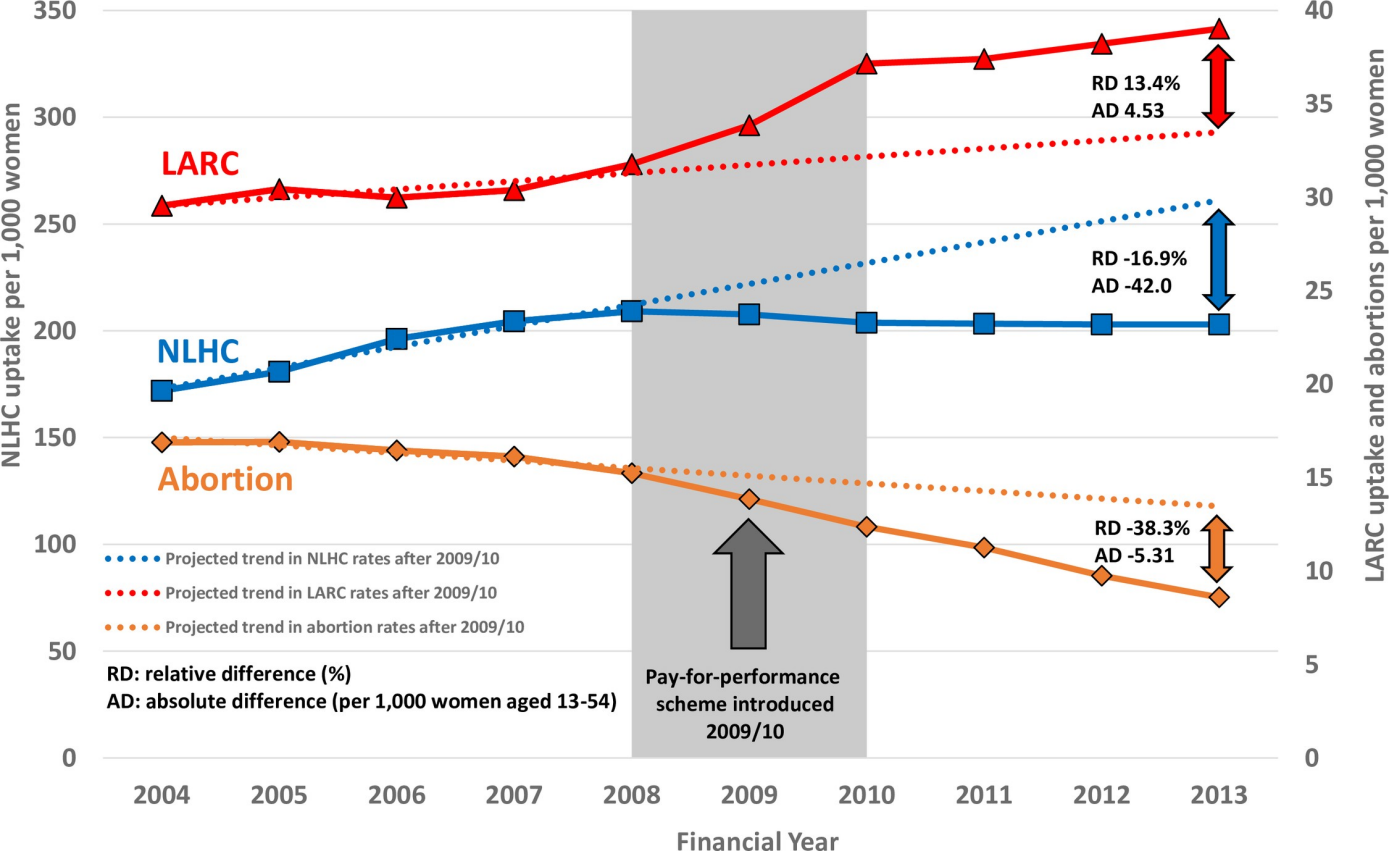
Summary figure. Trends in crude LARC and NLHC uptake and abortion rates between 2004/2005 and 2013/2014 in women aged 13–54 years in British primary care before and after introduction of P4P scheme for LARC advice. AD, absolute difference; LARC, long-acting reversible contraception; NLHC, non-LARC hormonal contraception; P4P, pay for performance; RD, relative difference.

Between 2004/2005 and 2013/2014, crude LARC uptake rates increased by 32.0% from 29.6 to 39.0 per 1,000 women, compared with a smaller increase (18.0%) from 172 to 203 per 1,000 women in NLHC uptake (Table 1 and Fig 1). Crude LARC uptake increased in all age groups from 2004/2005 to 2013/2014, but the relative change was greatest in women aged 35 to 54 years (48.1%), followed by 13 to 19 years (32.1%), 20 to 24 years (19.1%), and 24 to 34 years (18.6%). Crude LARC uptake was higher in women from most deprived than least deprived group throughout the study period, but relative increase in uptake was larger in least deprived (43.6%) compared with the most deprived (13.9%) group (Table 1, S5 Fig). Among the LARC methods, the contraceptive injection had the greatest uptake across the study period, but crude rates fell by 5.4% from 22.2 in 2004/2005 to 21.0 in 2013/2014; there was also a fall in IUD uptake by 8.5%. There was an 8-fold increase in crude uptake rate of contraceptive implants and a smaller rise in IUS uptake (86.0%) (S1B Table).

ITS analysis (Table 2) showed a small increase in trend (0.56, 0.54–0.59, *p <* 0.001) in LARC uptake rate in all ages before the P4P, and then an increase immediately after the P4P was introduced (5.36, 5.26–5.45, *p <* 0.001), followed by a small but sustained decrease in trend 4 years following the P4P (−0.35, −0.42 to −0.28, *p <* 0.001). All LARC methods had statistically significant and positive step changes in uptake immediately after the P4P; the greatest step changes occurred in injection (1.68, 1.67–1.70, *p <* 0.001) and implant (1.64, 1.63–1.65, *p <* 0.001). There was a reversal in the trend of contraceptive injection uptake from an initial decline pre-P4P (−0.86, −0.86 to −0.85, *p <* 0.001), step change increase (1.68,1.67–1.70, *p <* 0.001), followed by a sustained increase post-P4P (1.25, 1.24–1.27, *p <* 0.001).

**Table 2:** ITS analysis of trends in contraceptive uptake rate per 1,000 women in primary care in Britain before and after introduction of P4P scheme in 2009/2010.

		Pre-P4P trend	Step change	Post-P4P trend	Rate change/1,000 women 4 years after P4P	% change 4 years after P4P
**Contraceptive method**	**Age/deprivation group**					
LARC	All ages	0.56 (0.54 to 0.59)	5.36 (5.26 to 5.45)	−0.35 (−0.41 to −0.28)	4.53	13.4
Injection	All ages	−0.86 (−0.86 to −0.85)	1.68 (1.67 to 1.70)	1.25 (1.24 to 1.27)	5.83	38.0
Implant	All ages	0.71 (0.71 to 0.71)	1.64 (1.63 to 1.65)	−0.20 (−0.21 to −0.18)	1.57	24.8
IUD	All ages	−0.04 (−0.04 to −0.04)	0.21 (0.20 to 0.22)	0.00 (0.00 to 0.01)^1^	0.18	10.1
IUS	All ages	0.51 (0.51 to 0.52)	0.95 (0.95 to 0.96)	−0.54 (−0.55 to −0.54)	−0.70	−8.03
All LARC methods	13–19 y	0.15 (0.09 to 0.22)^2^	4.51 (4.18 to 4.83)	1.04 (1.00 to 1.08)	8.83	42.3
	20–24 y	−0.90 (−0.91 to −0.88)	8.40 (8.34 to 8.47)	3.14 (3.08 to 3.19)	20.1	45.1
	25–34 y	−0.50 (−0.50 to −0.49)	6.31 (6.28 to 6.34)	1.40 (1.37 to 1.42)	11.4	26.9
	35–54 y	0.99 (0.99 to 1.00)	2.27 (2.25 to 2.29)	−0.52 (−0.54 to −0.50)	1.19	4.01
	Least deprived	0.10 (0.10 to 0.10)	5.71 (5.70 to 5.71)	0.48 (0.48 to 0.48)	7.73	33.0
	Most deprived	−0.03 (−0.06 to −0.01)^3^	3.72 (3.61 to 3.83)	0.67 (0.58 to 0.76)	6.37	15.2
NLHC	All ages	9.25 (8.98 to 9.52)	−22.8 (−24.5 to −21.2)	−7.10 (−7.88 to −6.33)	−42.0	−16.9
CHC	All ages	5.24 (5.13 to 5.35)	−15.0 (−15.5 to −14.5)	−10.8 (−11.2 to −10.5)	−53.1	−28.7
POP	All ages	4.83 (4.81 to 4.85)	−2.60 (−2.69 to −2.51)	−0.33 (−0.37 to −0.30)	0.89	1.30
NLHC	13–19 y	8.45 (7.81 to 9.09)	−30.8 (−34.1 to −27.5)	−10.2 (−11.1 to −9.26)	−63.1	−23.4
	20–24 y	12.6 (11.9 to 13.4)	−42.7 (−46.8 to −38.7)	−17.6 (−19.2 to −16.1)	−101	−20.2
	25–34 y	14.7 (14.4 to 15.1)	−41.9 (−44.1 to −39.6)	−14.4 (−15.4 to −13.4)	−84.6	−20.5
	35–54 y	6.61 (6.51 to 6.70)	−10.3 (−10.8 to −9.80)	−3.48 (−3.75 to −3.21)	−17.6	−13.2
	Least deprived	11.5 (11.1 to 11.8)	−27.7 (−29.4 to −26.0)	−8.11 (−9.13 to −7.08)	−48.7	−18.7
	Most deprived	8.88 (8.42 to 9.34)	−30.6 (−33.0 to −28.3)	−9.77 (−11.2 to −8.35)	−60.8	−25.6

All *p* < 0.001 unless stated.

^1^*p* = 0.251.

^2^*p* = 0.004.

^3^*p* = 0.041.

**Abbreviations:** CHC, combined hormonal contraception; ITS, interrupted time series; IUD, intrauterine device; IUS, intrauterine system; LARC, long-acting reversible contraception; NLHC, non-LARC hormonal contraception; P4P, pay for performance; POP, progestogen-only pill.

There were statistically significant step increases in LARC uptake across all ages. The greatest rise occurred among women aged 20–24 years (8.40, 8.34–8.47, *p <* 0.001), with sustained increase in uptake in the 4 years after the introduction of the P4P (3.14, 3.08–3.19, *p <* 0.001). There was a small increase in the trend in LARC uptake in women from least deprived group pre-P4P (0.10, 0.10–0.10, *p <* 0.001), whereas the trend pre-P4P was decreasing in the most deprived group (−0.03, −0.06 to −0.01, *p =* 0.041). Immediately after the P4P there was greater step change in LARC uptake in the least deprived (5.71, 5.70–5.71, *p <* 0.001) compared with the most deprived group (3.72, 3.61–3.83, *p <* 0.001).

Our model estimated that 4 years after the P4P scheme, there were an additional 4.53 LARC prescriptions per 1,000 women, more than would have been expected had the P4P scheme not been introduced. This represents a relative increase of 13.4%.

### Trends in NLHC uptake

NLHC use was highest in women aged 20 to 24 years compared with other age groups and higher among women from the most affluent compared with the most deprived group (Table 1 and Fig 1). The rise in NLHC use across the study period was highest among women aged 35 to 54 years (44.1%) and more affluent women (24.7%) (S6 Fig).

The ITS analysis showed an increasing trend in NLHC uptake (9.25, 8.98–9.52, *p <* 0.001) pre-P4P, a step change of −22.9 (−24.5 to −21.2, *p <* 0.001), and a sustained decrease in trend of −7.10 (−7.88 to −6.33, *p <* 0.001) in NLHC uptake overall (Table 2). We observed a similar pattern among different NLHC methods (CHC and POP), all age groups, and deprivation quintiles.

By the end of the study period in 2013/2014, there was an overall fall in NLHC uptake of −42.0 per 1,000 women (−16.9%) compared with what would have been expected if the P4P scheme had not been introduced; this was mostly accounted for by falls in CHC uptake.

### Trends in abortion

Crude abortion rates fell by 49.2% from 16.9 to 8.59 across the study period (Table 1 and Fig 1); the biggest falls occurred in teenage women (−71.2% in those aged 13 to 19 years versus *−*31.7% in women aged 35 to 54 years S7 Fig). Falls in abortion rates were greater in women from least deprived compared with most deprived group (−59.0% versus −51.3%).

Trends in abortion rates in all ages were decreasing (Table 3) pre-P4P (−0.41, −0.55 to −0.27, *p =* 0.002); this was followed by a step change of −2.28 (−2.99 to −1.57, *p =* 0.002) immediately after the P4P was introduced and further sustained decreases in trends post-P4P (−0.88, −1.12 to −0.63, *p <* 0.001). There was an absolute reduction of −5.31 abortions per 1,000 women (relative reduction of −38.3%) in all ages compared with what would have been expected without the P4P scheme; this is equivalent to 95,170 additional abortions averted by 2013/2014 if extrapolated to the British population.

**Table 3:** Summary of ITS analysis of trends in abortion rates before and after introduction of P4P scheme in 2009/2010 in primary care practices in Britain.

Abortion rates per 1,000 women	Pre-P4P trend	Change in level	Post-P4P trend	Rate change 4 years after P4P	% change 4 years after P4P
**All ages**	−0.41 (−0.55 to −0.27)^1^	−2.28 (−2.99 to −1.57)^1^	−0.88 (−1.12 to −0.63)	−5.31	−38.3
**Age group (years)**					
13–19	−1.26 (−1.77 to −0.76)^2^	−5.04 (−7.56 to −2.51)^3^	−1.19 (−2.07 to −0.32)^4^	−9.87	−57.0
20–24	−2.07 (−2.65 to −1.49)	−4.52 (−7.48 to −1.57)^5^	−2.84 (−3.84 to −1.84)^6^	−18.0	−42.6
25–34	−0.16 (−0.39 to 0.07)^7^	−2.15 (−3.34 to −0.97)^8^	−1.54 (−1.95 to −1.14)	−8.49	−35.1
35–54	−0.09 (−0.14 to 0.04)^9^	−0.17 (−0.42 to 0.07)^10^	−0.13 (−0.22 to −0.04)^11^	−0.66	−19.2
**Least deprived**	−0.40 (−0.47 to −0.32)	−1.36 (−1.72 to −1.01)	−0.73 (−0.86 to −0.61)	−3.96	−44.0
**Most deprived**	−0.85 (−1.35 to −0.35)^12^	−4.40 (−6.89 to −1.91)^13^	−0.74 (−1.60 to 0.13)^14^	−7.46	−37.5

All *p* < 0.001 unless stated.

^1^*p* = 0.002.

^2^*p* = 0.005.

^3^*p* = 0.011.

^4^*p* = 0.044.

^5^*p* = 0.030.

^6^*p* = 0.003.

^7^*p* = 0.229.

^8^*p* = 0.016.

^9^*p* = 0.014.

^10^*p* = 0.227.

^11^*p* = 0.031.

^12^*p* = 0.020.

^13^*p* = 0.018.

^14^*p* = 0.156.

**Abbreviations:** ITS, interrupted time series; PHP, pay for performance

The greatest impact of the P4P policy on abortion rates occurred in women under 25 years. Women aged 13–19 had the largest step change (−5.04, −7.56 to −2.51, *p =* 0.011) followed by sustained decrease in trend of −1.19 (−2.07 to −0.32, *p =* 0.044) (Table 3). Women aged 20 to 24 years had step change of −4.52 (−7.48 to −1.57, *p =* 0.030) immediately after the P4P, followed by sustained decrease in trend in the 4 years post-P4P (−2.84, −3.84 to −1.84, *p =* 0.003). Abortion rates fell more among women from the most deprived group compared with those in the least deprived group immediately after the P4P (step change −4.40 versus −1.36). Abortion rates continued to fall post-P4P in all groups; the relative change in women from the least deprived group were greater (−44.0% least deprived versus −37.5% most deprived).

### Sensitivity analysis

Phase-in periods did not alter the trends of LARC uptake and abortion rates, but the association was greater with than without (S1C Table). For LARC uptake, the step change was 5.36 (5.26–5.45, *p <* 0.001) with the phase-in period and 3.34 (3.17–3.52, *p <* 0.001) without. The association with NLHC was greater; step change in NLHC was −22.9 (−24.5 to −21.2, *p <* 0.001) with phase in and −8.01 (−9.09 to −6.92, *p <* 0.001) without phase in. The step change in abortion rates associated with the P4P was greater with phase in (−2.28, −2.99 to −1.57, *p =* 0.002) than without (−1.49, −1.62 to −1.35, *p <* 0.001).

In our sensitivity analysis, we found a sustained reduction in abortion rates post-P4P under all 3 scenarios of missing data on abortion: 20%, 40%, or 50%, all of which were statistically significant (*p <* 0.001, S1D Table). Even if 50% additional abortion events were included in the analysis after the P4P, the trend post-P4P would have been −1.52 (1.78 to −1.26, *p <* 0001).

## Discussion

### Main findings

Our study showed a 13.4% increase in LARC uptake following the P4P scheme. This increase was unlikely to be related to a general increase in contraceptive uptake as we demonstrated a concomitant relative reduction of −16.9% in NLHC uptake in the same period. We also report a −38.3% reduction in the abortion rate compared with what would have been expected without the P4P scheme for LARC advice.

Young women aged 20 to 24 years increased their uptake of LARC the most, while those aged 13 to 19 years had the largest fall in abortion rates compared with other age groups. Women from the most deprived group had the largest decrease in abortion rates immediately following the P4P scheme. Overall, the P4P scheme had the biggest impact on younger women and those from the most deprived backgrounds.

### Comparison with current literature

The prevalence of contraceptive uptake in different age groups is consistent with findings from comparable surveys. The prevalence of oral contraceptive pill use in 2008/2009 was 54% in women aged 20–24 years and 10% in 40–49 years in one survey, compared with 44% (442 per 1,000 women) using NLHC among women aged 20–24 years and 11% (106 per 1,000 women) in women aged 35–54 years, respectively, in our study (S6 Fig) [21]. In a large probability sample survey conducted in 2010 to 2012, 26% of women aged 16 to 24 were using hormonal methods (defined in the study as patches, pills, and injections), and 11% used LARC (implant, IUD, IUS) [22]. In our study, age-specific NLHC rates in 2011/2012 were 21% for women aged 13 to 19 and 41% for women aged 20 to 24. Age-specific rates for LARC in 2011/2012 were 3% in women aged 13 to 19 and 6% in women aged 20–24 (S5 Fig). Our previous study examining P4P policy impact using dispensing data reported similar trends and patterns in individual LARC prescriptions [14].

Trends in abortion rates were similar to those from national surveillance records for England and Wales, which showed that the 20–24 age group had the highest crude age-specific rate (30.0 per 1,000 women) of all age groups in 2009; our data also showed this age group had the highest rates (44.6) among all ages in 2009/2010 (we used England and Wales data only for this comparison) [23]. The crude abortion rate in women under 18 years was 11.7 in 2013, which was a 33.5% reduction from 17.6 in 2009; our data showed a much greater reduction from 18.3 in 2009/2010 to 7.50 in 2013/2014 (59%) for ages 13 to 19 years (England and Wales data). Our study population of 3.2 million women with prospectively collected data is likely to provide a more accurate estimate than surveys based on self-reporting, and—as this is a population of women who engaged with primary care services in Britain—it would make sense that these women benefited more from interactions with their PCP.

### Strengths and limitations

To our knowledge, this is the first national evaluation of the impact of the contraceptive P4P scheme in Britain. Our large sample size from the CPRD database greatly enhances the representativeness of our findings to the British population and comparable middle- to high-income countries. Many previous studies on P4P schemes reported processes of care and not outcomes; even those that reported outcomes did not demonstrate long-term sustainability [24,25]. Ours is one of few that has demonstrated changes in both clinical process and patient outcomes in relation to one P4P scheme as well as sustainability for at least 4 years after its introduction. To strengthen our methodology, we used at least 3 time points before and after a clearly defined intervention period; we used an ARMA model for analysis; we conducted autocorrelation and sensitivity analysis; and we adhered to the recommended reporting framework for ITS studies to improve reporting and enable comparisons in systematic reviews (S2 File) [26].

However, there are important limitations of our study that relate to our design, accuracy, and completeness of data. The CPRD database might have a relatively good coverage of the population in Britain, but it is not a random sample: 5 out of 12 regions are overrepresented in the population, so there might be selection bias from the types of practices that contributed to the database (S3 Fig). Despite this, our population in 2004/2005 and 2013/2014 closely matched the age distributions of the mid-year UK census populations (S8 Fig). Another bias is that deprivation data were only available for the English population; we found a relatively larger number of women in the least deprived group (IMD 1), and this distribution of IMD was the same throughout the study period (S4 Fig). We cannot comment from our data and methodology on whether women from more deprived areas were targeted for LARC advice.

We only counted incident contraceptive prescriptions that might have underestimated NLHC methods as they had to be prescribed relatively more frequently due to their shorter duration of action; however, this was offset by the several-fold difference in volume of NLHC prescriptions compared with LARC. The use of prescription data also meant we could not draw conclusions about women who were sexually active but who were using methods that could not be prescribed, or nothing at all as they might be pregnant or trying to conceive.

Additionally, data completeness might be threatened by missing events and patients, misclassification, and miscoding of clinical events, especially abortions by PCPs; event data might be missing if abortion procedures were not reported back to the PCP. Nevertheless, we have demonstrated the prevalence of our outcome measures matched closely with those from other sources. The sensitivity analysis has demonstrated the reduction in abortion rates after the P4P scheme was still significant even if we added 50% additional events to the modelling.

There might also be missing data on NLHC and LARC as women obtain contraceptive care from sexual health clinics. While this might explain the low numbers of IUDs as more might be fitted in community contraceptive clinics, it would not explain the rise in IUSs or contraceptive implants. As we were interested in the impact of primary care interactions on contraceptive uptake and abortions, the main emphasis was on data recorded in primary care. There might be overestimation of IUSs because of their use in managing heavy menstrual bleeding; while this might have explained the higher prevalence of use in older women (just over 50% of our sample aged 35 to 54), it is unlikely to have had significant impact on the overall findings.

Scatter plots of crude rates before and after the interventions for all outcome measures suggested that linear modelling was the best approach, and this assumption was applied when calculating the difference between fitted models and the counterfactual. All predictive modelling is subject to a degree of uncertainty, so our predicted changes should be interpreted with caution.

Other interventions around the same time as introduction of the P4P could have affected validity of ITS analysis. Two important events might be relevant: firstly, national clinical guidance on LARC was published by National Institute for Health and Care Excellence (NICE) in 2005 at the start of the study period, which might have contributed to increasing awareness of LARC for clinicians and the public. Secondly, media campaigns to improve awareness of contraception and LARC among young people were introduced in November 2009; however, this only ran for a month, and it was unlikely that the impact lasted as long as the end of our study period in 2013/2014 [27]. In addition, the Teenage Pregnancy Strategy was introduced in England in 1999, and the policy was implemented for 10 years [28]; emergency hormonal contraception had been made available free of charge in Scotland from 2008 and in Wales in 2011, which could also contribute to reducing unplanned pregnancies [29,30]. However, our study design shows additional impact of the P4P scheme over underlying trends and in other age groups.

### Implications for policy and research

The rationale from policy makers for including LARC advice as part of the primary care P4P scheme was to ensure that women were informed of contraceptive choices to best meet their needs and also an assumption that “increasing the uptake of LARC methods will reduce the number of unintended pregnancies” [13]. The implications from our study suggest that offering financial incentives to PCPs to give LARC advice to women not only increased the uptake of LARC but also reduced abortion rates. The study outcome seemed to confirm our hypothesis. We speculate the mechanism for increasing LARC uptake might be that LARC advice from PCPs facilitated switching from NLHC to more reliable methods, such as LARC for some women, which in turn prevented more unintended pregnancies.

If these changes in contraceptive uptake and abortions occurred because of the P4P, we estimate that by 2013/2014, the P4P scheme resulted in an additional 4.53 LARC prescriptions per 1,000 women or relative increase of 13.4%, equivalent to 81,190 extra prescriptions if extrapolated to the British population. There was an absolute reduction of −5.31 abortions per 1,000 women, or −38.3% relative reduction, equivalent to 95,170 abortions averted if extrapolated to the British population.

There have been discussions in British primary care that the P4P scheme that has been running for over 15 years might be abolished [31]. The scheme attracted much discussion on the best ways to remunerate PCPs to deliver good quality care, what the intended consequences were, and the erosion of the patient’s agenda in the consultation [32–34]. Our study suggests there might be merit in retaining this scheme to support primary care to improve reproductive health outcomes. More recently, a study found that removal of some P4P indicators in British primary care was associated with decline in performance, quality, and documentation, including LARC advice [35]. An extension of our study for a longer period might yield answers regarding impact on reproductive outcomes—whether the effects on these might be sustained after removal of this P4P scheme.

As this is an ecological study, we are unable to comment on the causal pathway at the individual level. We are unable to comment about whether women switched from NLHC in favour of LARC because the latter were more desirable, more effective, safer (e.g., IUD is hormone-free, so reduced cardiovascular risks associated with oestrogens) or because they became better informed about options available to them. Future studies should examine the impact of LARC advice on contraceptive uptake and choice of individual women, which women responded to LARC advice, and the effect of LARC uptake on the risk of unintended pregnancy outcomes. There might also be policy differences among Scotland, England, and Wales that could be examined.

There are different opinions about what the best strategies should be at the population level to reduce unplanned and unwanted pregnancies. It has been estimated that more unintended pregnancies would be prevented by increasing the number of contraceptive users overall, rather than encouraging use of a specific methods [36,37]. Financial incentives, target-driven programmes, and strategies that focus on certain demographic subgroups might be effective but may be seen as coercive, particularly if LARC is promoted over other methods [38]. While we need to carefully consider women’s reproductive autonomy over promotion of LARC methods, these discussions do not diminish the importance of reducing barriers to LARC use for deprived populations [39].

At the individual level, LARC is not necessarily suitable for all women because it can have unwanted effects, and method preference will be based on a balance between reliability for long-acting methods and autonomy for short-acting methods. Some women may also use contraceptive methods for other indications, for example, some CHCs can be used to treat acne, IUS is licensed to treat heavy menstrual bleeding. The underlying principle of fertility control programmes should ensure women are fully informed about the range of contraceptive options so that they can make a choice fitting their needs and preferences and appropriate for their stage of life.

## Conclusions

Our study offers evidence that the P4P scheme to give LARC advice in primary care was followed by increased LARC uptake and falls in abortion rates. This scheme had greater impact on younger women and those from more deprived quintiles compared with all other groups.

## Supporting information

S1 File ISAC for MHRA database research (protocol number 15/076_R2). ISAC, Independent Scientific Advisory Committee; MHRA, Medicines and Healthcare Products Regulatory Agency
(PDF)

S2 File ITS study reporting checklist. ITS, interrupted time series. (PDF)

S1 Fig ITS regression model. ITS, interrupted time series. (TIF)

S2 Fig Denominator population by age groups. (TIF)

S3 Fig Denominator population by regions. (TIF)

S4 Fig Denominator population by deprivation groups. (TIF)

S5 Fig LARC rates by age and deprivation groups. LARC, long-acting reversible contraception.
(TIF)

S6 Fig NLHC rates by age and deprivation groups. NLCH, non-LARC hormonal contraception.
(TIF)

S7 Fig Abortion rates by age and deprivation groups. (TIF)

S8 Fig Comparison of age distributions between CPRD and UK Census population in years 2004 and 2013. CPRD, Clinical Practice Research Datalink.
(TIF)

S1 Table (A) LARC advice given as a percentage of eligible women aged 13 to 54 using an NLHC. (B) Uptake per 1,000 women aged 13 to 54 years by method of contraception 2004/2005 to 2013/2014. (C) Sensitivity analyses of outcomes with and without phase-in period. (D) Sensitivity analyses using scenarios of additional abortions. LARC, long-acting reversible contraception; NLHC, non-LARC hormonal contraception.
(PDF)

## Abbreviations

ARMA: autoregressive moving average
CHC: combined hormonal contraception
CPRD: Clinical Practice Research Datalink
GLS: Generalised Least Squares
IMD: Indices of Multiple Deprivation
ISAC: Independent Scientific Advisory Committee
ITS: interrupted time series
IUD: intrauterine device
IUS: intrauterine system
LARC: long-acting reversible contraception
MHRA: Medicines and Healthcare Products Regulatory Agency
NHS: National Health Service
NICE: National Institute for Health and Care Excellence
NLHC: non-LARC hormonal contraception
NRES: National Research Ethics Service
P4P: pay for performance
PAG: Project Advisory Group
PCP: primary care physician
POP: progestogen-only pills
