# Supplementary material for: Impact of a pay-for-performance scheme for long-acting reversible contraceptive (LARC) advice on contraceptive uptake and abortion in British primary care: An interrupted time series study
Source: PLoS Med. 2020 Sep 14;17(9):e1003333. doi: 10.1371/journal.pmed.1003333 (PMC7489538; doi:10.1371/journal.pmed.1003333)
Supplement: S1 File — ISAC, Independent Scientific Advisory Committee; MHRA, Medicines and Healthcare Products Regulatory Agency (PDF) [file pmed.1003333.s001.pdf]

Independent Scientific Advisory Committee (ISAC) for Medicines and Healthcare Products Regulatory Agency (MHRA) database research (protocol number 15/076_R2)

(Highlighted parts are the approved amendments)

What is the impact of financial incentives for UK general practitioners to offer advice on long acting reversible contraception on abortion rates and contraceptive uptake especially in teenage women?

Lay Summary (max.200 words)

General practitioners in the UK are incentivised to provide some clinical and non-clinical care through a pay-for-performance scheme called the Quality and Outcomes Framework (QOF). One of the requirements is to offer advice on long acting reversible contraception to women who use regular or request emergency hormonal contraception.

Long acting reversible contraception (LARC) includes: injections, implants and two types of contraception fitted inside the womb. They are reliable because they are not user dependent. They are also long lasting: contraceptive effects range from three months (injection) to ten years (copper intrauterine contraception). They are also good value for money for their duration of use compared with other non-LARC methods.

Despite these advantages the proportion of women in the UK who use LARC is low in comparison with other countries.

The incentive scheme for GPs has increased the uptake of LARC but we do not know what impact this has had on abortion and uptake of contraception, or indeed which groups or areas this has benefitted if at all. Improving the use of LARC may have an impact on teenage conception rates as well as overall rates of unplanned pregnancies.

The proposed research will examine: the impact of LARC advice on reproductive health outcomes and if this policy addressed health inequalities by benefitting women from more disadvantaged backgrounds who are at greater risk of abortions and unplanned pregnancies.

Background

Teenage pregnancies can have adverse effects on both the mother and baby. Three quarters of teenage pregnancies are unplanned and half end in an abortion. Teenage mothers and their children are at increased risk of poverty and poor health outcomes such as pre-term birth, low birth weight babies and higher rates of ill health.

Despite reductions in teenage pregnancy rate in the UK rates are higher compared with other western European countries; both conception and abortion follow the distribution of deprivation very closely. The teenage pregnancy strategy has been linked to the declines in teenage conceptions and birth rates and greatest in the areas receiving higher amounts of strategy-related funding.

Long acting reversible contraception (LARC) include: injections, implants and two types of contraception fitted inside the womb. These methods are reliable because they do not require the user to remember to take a pill every day. They are also long lasting: contraceptive effect range from three months (injection) to ten years (copper intrauterine contraception). LARC are very effective in reducing unplanned pregnancies. Teenagers are less likely to use contraception reliably so LARC can have an effect on reducing teenage pregnancies. However, the proportion of women

in the UK who use these methods for contraception are low in comparison to other countries. Improving the use of LARC may have an impact on teenage conception rates as well as overall rates in unplanned pregnancies.

Recently in the UK, several government initiatives have aimed to increase awareness and use of LARC including public policy initiatives such as National Institute for Health and Clinical Excellence (NICE) LARC guidelines and Quality and Outcome Framework (QOF). The QOF scheme was implemented in April 2004 by the National Health Services (NHS), linked GP income to performance against targets set in QOF indicators.

A new set of Quality and Outcome Framework (QOF) indicators on contraception was introduced in the 2009-10 General Medical Services (GMS) contract for General Practices. Unlike previous years' QOF indicators in sexual health (CON1 and CON2) where the focus was only on having a policy for emergency contraception requests and provision of pre-conceptual advice. Sexual health indicators (SH1, SH2, SH3) introduced in April 2009 focused on provision of information on long acting reversible methods of contraception to women (under the age of 54) attending for contraceptive advice aimed at increasing awareness of LARC methods among women seeking contraceptive advice in general.

GPs are only incentivised for structural and process aspects of contraceptive provision, i.e. having a policy on contraception and giving out advice respectively. We do not know if this has had impact on women and their use of contraception, and subsequent reduction in unplanned pregnancies and abortion rates.

Rationale

Our previous ecological study revealed incentivising GPs to provide LARC has increased its uptake in primary care but little is known about how the policy has impacted on reproductive health outcomes such as pregnancy and abortion, and which groups or areas, if any, it has benefitted or to whom it could be extrapolated.

We plan to use a cohort of women of reproductive age (13 to 54 years – age range used in QOF indicator) registered with a GP in CPRD dataset and follow through their contraceptive use and pregnancy outcomes in the period of study. The incentive for LARC advice was introduced for GPs in 2009 so we will consider up to 5 years before this time and prospectively the outcomes to date. This will mean at least 10 years' worth of follow up from 2004 to 2014. We will use abortion as an end point and proxy marker for unplanned and unwanted pregnancy.

This practice-level analysis will see if practices with higher achievements for providing LARC advice have higher uptake rates of LARC and lower abortion rates.

We also plan to explore whether the policy has been equitably achieved and its impact on women who are at increased risk of teenage pregnancy and abortion including those from disadvantaged backgrounds and certain minority ethnic groups.

Our hypothesis is that the policy has not benefitted women who are most at risk for abortion such as younger women and women who are not already on regular hormonal contraception. As the QOF advice is only given for women using non-LARC hormonal contraception or those requesting

emergency contraception, this may suggest a cohort of women with unmet needs (i.e. those who did not get regular hormonal contraception or emergency contraception from their GPs.

Objective, specific aims

Aim:

To investigate the impact of pay-for-performance incentive schemes [i.e. Quality and Outcomes Framework (QOF)] for UK general practitioners to improve uptake of long-acting reversible contraception on contraceptive use and abortion in women aged 13-54 years

Objectives

The analysis will be at three levels – individual, practice and health systems

1. ~~At the individual level, we will conduct an analysis of contraception uptake, LARC uptake and abortion rates in women of reproductive age.~~
2. ~~At the practice level – we will conduct analysis to correlate practices' LARC advice with abortion rates~~
3. ~~At the health systems/ecological level, analyse the impact of LARC advice which commenced in 2009 in general practice on abortion rates and LARC use~~
1. At the individual level, we will conduct an analysis of contraception uptake, LARC uptake and abortion rates in women of reproductive age.
2. At the practice level – we will conduct analysis to correlate practices' LARC advice with abortion rates
3. At the health systems/ecological level, analyse the impact of LARC advice which commenced in 2009 in general practice on abortion rates and LARC use

Study Type

Observational cohort study

Study Design

Retrospective cohort study following up women of reproductive age (13 to 54) for a period of 5 years before (2004 to 2009) and 5 years after (2009 to 2014) the introduction of financial incentives for LARC advice in general practice (financial year 2009/10)

Sample size/power calculation

We anticipate an increase of LARC use by 15%. Using the lowest prevalence of LARC use which is 1 per 1000 for intrauterine device (IUD) in women of reproductive age. Sample size calculation using 95% confidence interval and power of 0.8 = 762, 472 women.

We understand from informal discussion with CPRD Knowledge Centre that there are approximately 30,000 events coded as "L05.00 - Legally induced abortion".

According to the latest abortion statistics from Department of Health, the age standardised abortion rate was 15.9 per 1000 resident women aged 15-44.

To get a 15% reduction at 5% significance and power of 0.8, the number of women needed for analysis would be 47,198

Study population

(including estimate of expected number of relevant patients in the CPRD)

Case definition:

- Women of reproductive age from 13 to 54 who entered the cohort between 2004 and 2014 who were on non-LARC contraception and given LARC advice

Also include

- Women who are pregnant – we will include this group because we assume they would require contraception within one year of that code being entered – which would usually include the time period where a postnatal check would be done and at theoretical risk of pregnancy.
- We will also include women who were coded as having "termination counselling" or "referred for TOP" but without subsequent code for abortion. This represents a group of women who might have been ambivalent about pregnancy and thus might have benefitted from contraception

- Exclude:
    - women who are trying to get pregnant, under fertility treatment
    - women who has been sterilised or had hysterectomy
    - partner sterilised (so no need for contraception)
    - menopause

Selection of comparison group(s) or controls

- Women aged 13 to 54 years who entered the cohort between 2004 and 2014 who were using non-LARC method of contraception (barrier, oral contraceptive pill, vaginal ring, contraceptive patch)
- Women who were not known to be using any method of contraception.

Exposures, outcomes and covariates

Exposures

- LARC advice given by healthcare professionals in general practice (see Read code list)

Outcomes

- Abortion (primary outcome)
- LARC use (secondary outcome – prescription data or Read code list)
- Discontinuation of LARC (secondary outcome – Read code list included)

Covariates

- Age
- Deprivation
- IMD
- ~~Ethnicity~~

Follow up period:

- From 2004 to 2014 (5 years before and after 2009 when QOF marker for LARC advice was introduced)
- End of follow period – left practice/migration, death.

Use of linked data

We wish to ascertain adverse birth outcomes (i.e. abortion) and the equity of contraceptive provision.

HES data

We will use HES data to look for legally induced abortion procedures including surgical abortion and early medical abortion.

The abortion data from HES could be linked with those on CPRD in order to validate abortion codes from CPRD, or provide outcome information for women in cohort e.g. for those not using contraception, failed contraception, those referred for abortion but no subsequent coding of abortion procedure or antenatal care.

Abortion procedures in HES might also be a result of referrals from other sources such as community family planning clinics. This may provide information on another group of women who might have unmet needs.

IMD

We will use IMD to consider the equity of contraception provision, particularly if the incentive given to general practitioners to offer advice on long acting reversible contraception benefited all women from all backgrounds. We will also use IMD to consider if women from more disadvantaged backgrounds are over represented in abortion statistics.

IMD will be based on the woman's place of residence rather than post code of the GP practice they are registered with.

Data/ Statistical Analysis Plan

- ~~Risk Ratio of abortion in women not using contraception, using hormonal contraception prescribed by GP and those using LARC; also comparing women of different age bands to compare effect on under 18s compared with other age groups.~~
- ~~Odds ratio of abortion comparing women in above groups~~
- ~~Logistical regression looking at abortion, continuing use of contraception according to age, IMD, ethnicity~~
- ~~Survival analyses – abortion, length of use of LARC and non-LARC contraception~~

- ~~Cox proportional hazards model comparing length of use of different methods in women of different demographics and backgrounds~~

We propose to analyse the data as follows:

1. At the ecological level – to investigate the impact of LARC advice on abortion rates and LARC use
   - Changes in proportions of women using LARC and undergoing abortion before and after QOF indicator for LARC advice was introduced in 2009
   - We will look at differences in rate ratios between the two periods (pre 2009 and post 2009, as well as between two cohorts of women (under 18 and over 18).

2. At the practice level – to investigate the impact of QOF achievement in giving LARC advice on the prevalence of LARC usage
   - We will generate categories of QOF achievement e.g. 70-79%, 80-89%, 90%+
   - Linear regression to correlate practices' QOF achievement on LARC advice with abortion rates and LARC usage

3. At the individual level:
   - survival analysis, with mode of contraception and LARC advice as time-varying covariates and a repeated events framework
   - Multi-state model to calculate the transition rate between different modes of contraception.
   - Multi-state modes are useful when we want to know the transition rate (or related quantities like probability of transition and mean sojourn time) between different states (e.g., illness, recovery and death). The states in this project relate to the type of contraception used and the pregnancy outcomes of interest i.e. abortion.

- STATA 11 will be used for statistical analysis

There is plan for addressing confounding

- Use of logistic regression to adjust for deprivation - Index of Multiple Deprivation (IMD), age and ethnicity
- We can use logistic regression to adjust for practices that are paid to provide LARC service such as fitting of intrauterine contraception and contraceptive implants
- There is a plan for addressing missing data
- Sources of missing data –
  - Women who were seen in community contraceptive and sexual health clinics, pharmacists for contraception.
  - Women who had abortion in third sector providers e.g. Marie Stopes International, British Pregnancy Advisory Service
- Use sensitivity analysis to compare findings from this study with other surveys e.g. NATSAL and ONS data, estimating proportion of women who might avoid unplanned and unwanted pregnancy

Patient/ user group involvement †

No

Limitations of the study design, data sources and analytic methods

- ~~Not all birth outcomes are coded or collected by CPRD~~
- ~~Missing data~~
- ~~Some women attend other venues to obtain contraception so if no contraception issued or coded in GP does not mean not using contraception~~
- ~~Attrition and lost to cohort~~
- ~~Some practices excluded from CPRD~~
- ~~Female GP bias~~
- ~~As with any ecological study design evaluating the changes of a national policy the changes observed in the time frame against the background trends may in part be explained by the effect of concurrent efforts to increase LARC for example as a result of social marketing eg "Sex – worth talking about" campaign in 2009 http://www.nhs.uk/sexualhealthprofessional/Documents/Sex_Worth_Talking_About_brand_guidelines.pdf~~

Bias

- CPRD practices may not be representative of typical practice
- May be a difference in practices which code abortion vs practices which do not
- Women who obtain contraception and LARC from CASH clinics
- Women who had abortions in community clinics not coded in CPRD or noted in HES
- There may be larger number of women using LARC in practices that are participating in pay-for-service to fit intrauterine contraception or contraceptive implants.

Confounding

- Practices with female GPs may be more likely to give LARC advice and use LARC e.g. availability of skills in practice to fit IUD/implants
- Practices which provide LARC service more likely to have women on LARC
- LARC uptake may not be explained by QOF marker alone
- Other factors for increase/decrease in abortions or contraceptive use eg "pill scare"
- As with any ecological study design evaluating the changes of a national policy the changes observed in the time frame against the background trends may in part be explained by the effect of concurrent efforts to increase LARC for example as a result of social marketing eg "Sex – worth talking about" campaign in 2009

Missing data

- CPRD/HES mismatch abortion procedures

- There may be poor recording of ethnicity data prior to 2006 on CPRD. We anticipate that we may need to impute missing ethnicity assuming that ethnicity is missing at random. To impute we will carry out multiple imputation (MI), an approach used to deal with missing data. It can handle >50% of data missing at random and produce unbiased parameter estimates when used appropriately. We intend to use a full conditional specification approach and to carry out sensitivity analysis after MI to check assumptions.

  Sensitivity analysis using national data eg NATSAL, HSCIC

Sensitivity and confidentiality

There may be a possibility of unintentional (deductive) disclosure which may arise when there are small numbers of patients quoted. We will not report on data on sensitive information such as abortion with fewer than 5 events. Data were there are fewer than 5 events will be reported as a phrase "*5 or fewer*" rather than the number.

Plans for disseminating and communicating study results

- Conference presentation (oral or poster)
- Publication in a peer reviewed journal
- Once accepted for publication, Press release
- Social media such as Twitter
- Speaking at educational events and conferences that relate to teenage pregnancy, general reproductive and sexual health, contraception

See attached Flowchart PDF

READ CODE LIST (this list is not exhaustive)

Read code list of exclusions

**6122**	**12999**	**Not sexually active**
**6123**	**13001**	**No partner at present**
**6124**	**1328**	**Partner had vasectomy**
**6124.11**	**22934**	**Partner sterilised**
**6125**	**12996**	**Trying to conceive**
**6125.11**	**12997**	**Planning to start family**

Hysterectomy

**1599**	**6231**	**H/O: hysterectomy**
**685H.00**	**12920**	**No smear - benign hysterectomy**
**685H.11**	**12910**	**No smear - hysterectomy**
**685I.00**	**12930**	**No smear-amputation of cervix**
**685J.00**	**12918**	**Vaginal vault smear due**
**685K.00**	**12922**	**No smear - no cervix**

**7E03y00**	**42118**	**Other specified other operation on cervix uteri**
**7E03z00**	**34964**	**Other operation on cervix uteri NOS**
**7E04.00**	**9735**	**Abdominal excision of uterus**
**7E04.11**	**2448**	**Abdominal hysterectomy**
**7E04.12**	**3064**	**Wertheim hysterectomy**
**7E04000**	**35264**	**Abdominal hysterocolpectomy and excision periuterine tissue**
**7E04100**	**31312**	**Abdominal hysterectomy & excision of periuterine tissue NEC**
**7E04200**	**39748**	**Abdominal hysterocolpectomy NEC**
**7E04300**	**269**	**Total abdominal hysterectomy NEC**
**7E04311**	**69607**	**Bonney abdominal hysterectomy**

**7E04312**	**3666**	**Hysterectomy NEC**
**7E04400**	**1729**	**Subtotal abdominal hysterectomy**
**7E04500**	**813**	**Abdominal hysterectomy and bilateral salpingoophorectomy**
**7E04511**	**23863**	**Abdominal hysterectomy & bilateral salpingoophorectomy (BSO)**
**7E04512**	**7798**	**TAH - total abdom hysterectomy & bilateral salpingoophorect**
**7E04600**	**19182**	**Radical hysterectomy**
**7E04700**	**1830**	**Abdominal hysterectomy and right salpingoopherectomy**
**7E04711**	**2058**	**Abdominal hysterectomy and left salpingoopherectomy**
**7E04800**	**7949**	**Abdominal hysterectomy and left salpingoophorectomy**
**7E04900**	**3433**	**TAH - Tot abdom hysterectomy and BSO - bilat salpingophorect**
**7E04A00**	**11662**	**Abdominal hysterectomy with conservation of ovaries**
**7E04B00**	**25815**	**Lapar total abdominal hysterect bilat salpingo-oophorectomy**
**7E04C00**	**18980**	**Laparoscopic hysterectomy**
**7E04D00**	**96162**	**Excision of accessory uterus**
**7E04E00**	**94549**	**Laparoscopic subtotal hysterectomy**
**7E04F00**	**94934**	**Subtotal abdominal hysterectomy with conservation of ovaries**
**7E04G00**	**94490**	**Total abdominal hysterectomy with conservation of ovaries**
**7E04y00**	**14655**	**Other specified abdominal excision of uterus**
**7E04z00**	**15056**	**Abdominal excision of uterus NOS**
**7E05.00**	**24101**	**Vaginal excision of uterus**
**7E05.11**	**52057**	**Schauta radical vaginal hysterectomy**
**7E05.12**	**873**	**Vaginal hysterectomy**
**7E05000**	**59569**	**Vaginal hysterocolpectomy and excision of periuterine tissue**
**7E05100**	**54109**	**Vaginal hysterectomy and excision of periuterine tissue NEC**
**7E05200**	**28429**	**Vaginal hysterocolpectomy NEC**
**7E05300**	**7441**	**Vaginal hysterectomy NEC**
**7E05311**	**100097**	**Heaney vaginal hysterectomy**
**7E05400**	**19088**	**Laparoscopic vaginal hysterectomy**
**7E05500**	**42949**	**Vaginal hysterectomy with conservation of ovaries**
**7E05600**	**97020**	**Lap assist vag hysterectomy with bilat salpingo-oophorectomy**
**7E05y00**	**48866**	**Other specified vaginal excision of uterus**

7E05y11	47215	Ward vaginal hysterectomy
7E05z00	38349	Vaginal excision of uterus NOS

Sterilisation

6841	Open bilateral female sterilisation
14653	Open bilateral ligation of fallopian tubes
35984	Pomeroy open bilateral ligation of fallopian tubes
8007	Open bilateral clipping of fallopian tubes
60735	Open bilateral ringing of fallopian tubes
50773	Other specified open bilateral occlusion of fallopian tubes
4935	Open bilateral occlusion of fallopian tubes NOS
45721	Other open occlusion of fallopian tube
7163	Other open female sterilisation

Fertility treatment

8C82.00	33458	Female infertility therapy

K5B..00 1808	Infertility - female	
K5B0.00	4977	Female infertility of anovulatory origin
K5B0.11	16360	Anovular cycle
K5B0000	52132	Primary anovulatory infertility
K5B0100	50116	Secondary anovulatory infertility
K5B0z00	63421	Female infertility of anovulatory origin NOS
K5B1.00	69884	Female infertility of pituitary - hypothalamic origin
K5B1000	94448	Primary pituitary - hypothalamic infertility
K5B1100	99535	Secondary pituitary - hypothalamic infertility
K5B1z00	62084	Female infertility of pituitary - hypothalamic cause NOS
K5B2.00	35074	Female infertility of tubal origin
K5B2000	60861	Primary tubal infertility
K5B2100	45985	Secondary tubal infertility

**K5B2300**	**8352**	**Blocked fallopian tube**
**K5B2z00**	**25077**	**Female infertility of tubal origin NOS**
**K5B3.00**	**61299**	**Female infertility of uterine origin**
**K5B3000**	**91280**	**Primary uterine infertility**
**K5B3100**	**73151**	**Secondary uterine infertility**
**K5B3z00**	**68664**	**Female infertility of uterine origin NOS**
**K5B4.00**	**62698**	**Female infertility of cervical origin**
**K5B4000**	**97461**	**Primary cervical infertility**
**K5B4100**	**69324**	**Secondary cervical infertility**
**K5B5.00**	**48461**	**Female infertility of vaginal origin**
**K5B5100**	**96463**	**Secondary vaginal infertility**
**K5B6.00**	**54282**	**Female infertility associated with male factors**
**K5B7.00**	**104569**	**Female infertility due to diminished ovarian reserve**
**K5By.00**	**36458**	**Other female infertility**
**K5By000**	**2014**	**Primary infertility unspecified**
**K5By100**	**1943**	**Secondary infertility unspecified**
**K5Byz00**	**53018**	**Other female infertility NOS**
**K5Byz11**	**7246**	**Subfertility**
**K5Bz.00**	**30392**	**Female infertility NOS**

Menopause induced or otherwise

1512 4383 Menopause

**C162.00**	**73041**	**Postablative ovarian failure**
**C162000**	**102275**	**Postsurgical ovarian failure**
**C162100**	**50462**	**Postirradiation ovarian failure**
**C162200**	**93791**	**Other iatrogenic postablative ovarian failure**
**C163.00**	**3686**	**Other ovarian failure**
**C163.11**	**23802**	**Ovarian hypogonadism**
**C163000**	**31030**	**Primary ovarian failure**

**C163100**	**15992**	**Secondary ovarian failure**
**C163111**	**2087**	**Premature menopause NOS**
**C163200**	**31274**	**Hypergonadotrophic ovarian failure**
**C163300**	**22836**	**Ovarian hypogonadism**
**C163400**	**94499**	**Early menopause**
**C163y00**	**40672**	**Other specified other ovarian failure**
**C163z00**	**15075**	**Other ovarian failure NOS**

Read code list for Long Acting Reversible Contraception advice

**8CAw.00**	**96915**	**Advice about long acting reversible contraception**
**8CAW.00**	**68325**	**Patient advised to have pregnancy test**
**8CAw000**	**101024**	**Advice about IUCD, checking for threads**
**8CAw100**	**102309**	**Verbal advice about long acting reversible contraception**
**8CAw200**	**102308**	**Written advice about long acting reversible contraception**

**8CEG.00**	**96937**	**Long acting reversible contraception leaflet given**

Read code list for LARC contraception

**61B..00**	**12992**	**Depot contraceptive**
**61B..11**	**11507**	**Depot contraception**

**61B1.00**	**19506**	**Depot contraceptive given**
**61B1.11**	**8141**	**Depo-provera injection given**
**61B2.00**	**22938**	**Depot contraceptive repeated**
**61B3.00**	**22940**	**Depot contraceptive-no problem**

**7E09.00**	**6772**	**Intrauterine contraceptive device procedure**
**7E09.11**	**21114**	**Coil intrauterine contraceptive device procedure**
**7E09.12**	**17440**	**Intrauterine device procedure**
**7E09000**	**6941**	**Introduction of intrauterine contraceptive device**

7E09011 9226 Fitting of intrauterine contraceptive device

7E09100 22652 Replacement of intrauterine contraceptive device

7E09111 6050 Change of intrauterine contraceptive device

7E09400 7255 Introduction of Mirena coil

7E09500 18745 Removal of Mirena coil

7E09600 106401 Replacement of intrauterine system

7E09y00 53523 Other specified intrauterine contraceptive device

7E09z00 26317 Intrauterine contraceptive device procedure NOS

962..00 32870 FP1002 status

962..11 40402 Coil contraceptive claim

962..12 55297 FP1002 - IUD insertion claim

962..13 27872 IUCD contraceptive claim

962..14 27859 IUD contraceptive claim

962..15 99571 IUD contraceptive claim

962Z.00 25864 FP1002 status NOS

615..00 4049 Intra-uterine contr. device

615..11 10614 Coil contraception

615..12 17573 IUD contraception

615F.00445 IUD check

615G.00 225 IUD in situ

615K.00 22914 Intrauterine contraceptive device annual review

615L.0047908 Intrauterine contraceptive device 6 week check

615M.00 52230 Intrauterine contraceptive device annual review by telephone

615N.00 88224 Intrauterine contraceptive device fit by another GP practice

615P.00 94948 IUCD fitted by other healthcare provider

615P000 107463 Hormone releasing IUCD fitted by other healthcare provider

615Q.00 95476 Intrauterine contracep device removed by other hlth provider

615R.00 95906 Intrauterine contracep device checked by other hlth provider

615S.00 98121 Mirena coil check

615T.00 104471 Intrauterine contraceptive device threads seen

615Z.00 2795 IUD - NOS

6132 2738 IUD in situ from other agency

615..00 4049 Intra-uterine contr. device

615..11 10614 Coil contraception

615..12 17573 IUD contraception

ZV25414 20557 [V]Intrauterine contraceptive device check

ZV25415 31602 [V]Reinsertion of intrauterine contraceptive device

ZV25100 17980 [V]Intrauterine contraceptive device insertion

ZV25111 6064 [V]Coil insertion

ZV25112 2144 [V]Intrauterine contraceptive device insertion

ZV25113 3882 [V]Intrauterine contraceptive device insertion

ZV25411 20658 [V]Coil check

ZV25412 21365 [V]Reinsertion of coil

61F2.0043242 'Morning after' IUCD fitted

61A2.00 38544 'Morning after' IUD fitted

61A2.11 42310 Post-coital IUD fitted

6151100 102545 Insertion of T shaped 375 mm squared copper coated IUCD

98BA.00 71434 GMS3 claim - temporary contraceptive (IUCD) signed

98BB.00 72078 GMS3 claim - temporary contraceptive (IUCD) sent to HA

98BC.00 99268 GMS3 claim - temporary contraceptive (IUCD) paid

98CK.00 61591 GMS4 claim - contraception (IUCD) signed

98CL.0068735 GMS4 claim - contraception (IUCD) sent to HA

98CM.00 97329 GMS4 claim - contraception (IUCD) due with new IUCD

9kA0.00 61445 IUCD fitting - enhanced service completed

ZV25419 6265 [V]Intrauterine contraceptive device check

61R..00 102368 Intrauterine system contraception

9kr..00 103004 Subdermal etonogestrel implant insertion ESA

7G2AA00 11316 Insertion of Norplant

964..00 Insertion of subcutaneous contraceptive claim

61K..00 26092 Subcutaneous contraceptive

61KA.00 22950 Insertion of subcutaneous contraceptive

61KB.00 26477 Check of subcutaneous contraceptive

61KC.00 100958 Insert subcutaneous contraceptive implnt othr healthcre prov

61KD.00 93404 Subcutaneous contraceptive in situ

61KE.00 96963 Subcutaneous contraceptive implant palpable

61KH.00 106666 Subcutaneous contraceptive implant not palpable

61KZ.00 22951 Subcutaneous contraceptive NOS

7G2AG00 96953 Insertion of Implanon

7G2AH00 101819 Reinsertion of subcutaneous contraceptive

7G2AJ00 103642 Insertion of etonogestrel radiopaque contraceptive implant

964..00 104317 Insertion of subcutaneous contraceptive claim

Read for discontinuation of LARC

61B5.00 22947 Depot contraception stopped

61B4.00 29030 Depot contraceptive - problem

61B5.00 22947 Depot contraception stopped

6152 446 IUD removed

615Q.00 Intrauterine contracep device removed by other hlth provider

615B.00 22946 IUD expelled

615B.11 22945 IUD fallen out

615C.00 23421 IUD failure - pregnant

615C.11 29692 Pregnant, IUD failure

615D.00 30765 IUD partially expelled

ZV25413 20424 [V]Removal of coil

61KF.00 101010 Remov subcutaneous contraceptive implant othr healthcre prov

7G2H500 8153 Removal of Norplant

7G2H700 26260 Removal of subcutaneous contraceptive

965..00 104861 Removal of subcutaneous contraceptive claim

7G2HA00 96962 Removal of Implanon

7G2HB00 103620 Removal of etonogestrel radiopaque contraceptive implant

7G2HC00 107048 Removal of subcut contraceptive implant using US guidance

7G2H500 8153 Removal of Norplant

7G2H600 22875 Removal of hormone implant from subcutaneous tissue

7G2H700 26260 Removal of subcutaneous contraceptive

Read codes for non-LARC hormonal contraception

6141 19499 Oral contraceptive started

6142 15255 Oral contraceptive stopped

6143 19508 Oral contraceptive re-started

6144 12993 Oral contraceptive repeat

6145 13007 Oral contraception -no problem

6146 20581 Oral contraception - problem

6147 20354 Combined oral contraceptive

6148 2438 Progestagen only oral contrac.

6148.11 19505 Mini-pill: oral contraceptive

6148.12 12994 Progestagen only pill

6148.13 104174 Progestogen only oral contraceptive

6148.14 104177 Progestogen only pill

6149 20360 Oral contraceptive changed

614..00 12995 Oral contraceptive

614..11 5666 Oral contraception

614..12 6358 Pill - oral contraception

614A.00 19502 OCP for non-contraceptive use

614B.00 19504 Combined O.C. pill failure

614C.00 19503 Progestogen-only Pill failure

614D.00 180 Oral contraceptive prescribed

614E.00 2282 Oral contraceptive advice

614E.11 2122 Pill check

614F.006477 Emergency contraception advice

614Z.00 19501 Oral contraception NOS

ZV25015 8387 [V]Oral contraceptive prescription

ZV25417 20716 [V]Repeat prescription of oral contraceptive

ZV25418 37428 [V]Repeat prescription of oral contraceptive (OC)

61A..11 6635 Morning-after pill

61A1.00 715 'Morning after' pills given

61A1.11 26475 Post coital pills given

Read codes for abortion

L05..0A76259:C763290 Legally induced abortion

L05..11 Elective abortion

L05..12 Termination of pregnancy

L05..13 Therapeutic abortion

L050.00Legal abortion unspecified

L050000 Unspecified legal abortion + genital tract/pelvic infection

L050100 Unspecified legal abortion + delayed/excessive haemorrhage

L050200 Unspecified legal abortion + damage to pelvic organs/tissues

L050w00 Unspecified legal abortion with other specified complication

L050x00 Unspecified legal abortion with complication NOS

L050y00 Unspecified legal abortion with no mention of complication

L050z00 Unspecified legal abortion NOS

L051.00Legal abortion incomplete

L051.11Medal abortion - incomplete

L051.12Surgical abortion - incomplete

L051000 Incomplete legal abortion + genital tract/pelvic infection

L051100 Incomplete legal abortion + delayed or excessive haemorrhage

L051700 Incomplete medical abortion

L051711 Incomplete termination of pregnancy

L051w00 Incomplete legal abortion with other specified complication

L051y00 Incomplete legal abortion with no mention of complication

L051z00 Incomplete legal abortion NOS

L052.00Legal abortion complete

L052.11Medical abortion - complete

L052.12Surgical abortion - complete

L052000 Complete legal abortion + genital tract or pelvic infection

L052100 Complete legal abortion with delayed/excessive haemorrhage

L052500 Complete legal abortion with shock

L052w00 Complete legal abortion with other specified complication

L052x00 Complete legal abortion with complication NOS

L052y00 Complete legal abortion with no mention of complication

L052z00 Complete legal abortion NOS

L05z.00 Legally induced abortion NOS

L06..00 Illegally induced abortion

L06..11 Criminal abortion

L06..12 Self-induced abortion

L060.00Illegal abortion unspecified

L060z00 Unspecified illegal abortion NOS

L061.00Illegal abortion incomplete

L061z00 Incomplete illegal abortion NOS

L062.00Illegal abortion complete

L062000 Complete illegal abortion + genital tract/pelvic infection

L062w00 Complete illegal abortion with other specified complication

L06z.00 Illegally induced abortion NOS

L07..00 Unspecified abortion

L070.00 Unspecified abortion

L070300 Unspecified abortion with renal failure

L070x00 Unspecified abortion with complication NOS

L070z00 Unspecified abortion NOS

L071.00 Unspecified abortion incomplete

L071100 Unspecified incomplete abortion + delayed/excess haemorrhage

L071500 Unspecified incomplete abortion with shock

L071y00 Unspecified incomplete abortion + no mention of complication

L071z00 Unspecified incomplete abortion NOS

L072.00 Unspecified abortion complete

L072y00 Unspecified complete abortion + no mention of complication

L072z00 Unspecified complete abortion NOS

L07z.00 Unspecified abortion NOS

L08..00 Failed attempted abortion

L08x.00 Failed attempted abortion with complication NOS

L08y.00 Failed attempted abortion with no mention of complication

L08z.00 Failed attempted abortion NOS

ZV25311 [V]Admission for termination of pregnancy (TOP)

ZV25313 [V]Admission for termination of pregnancy

7E06600 Hysterotomy and termination of pregnancy

7E07113 Curettage of uterus for termination of pregnancy NEC

7E07114 Curettage of uterus for termination of pregnancy NEC

7E08400 Suction termination of pregnancy

7E08411 Vacuum termination of pregnancy

7E08500 Dilation of cervix and extraction termination of pregnancy

7E08600 Termination of pregnancy NEC
