# Supplementary material for: Impact of a pay-for-performance scheme for long-acting reversible contraceptive (LARC) advice on contraceptive uptake and abortion in British primary care: An interrupted time series study
Source: PLoS Med. 2020 Sep 14;17(9):e1003333. doi: 10.1371/journal.pmed.1003333 (PMC7489538; doi:10.1371/journal.pmed.1003333)
Supplement: S2 File — (PDF) [file pmed.1003333.s002.pdf]

Interrupted time series study reporting check list

Adapted from :Jandoc R, Burden AM, Mamdani M, Lévesque LE, Cadarette SM. Interrupted time series analysis in drug utilization research is increasing: systematic review and recommendations. Journal of Clinical Epidemiology. 2015;68(8):950-6. doi: 10.1016/j.jclinepi.2014.12.018.

**Item**		**No**	**Recommendation**	**Section & para no**
Title and abstract		1	Indicate the study design (interrupted time series) in the title or abstract	Title
Introduction	Background/ rationale	2	Provide background regarding the intervention and setting under investigation to support the study rationale and methods	Introduction 1-3
	Objectives	3	(a) State specific objectives and any prespecified hypotheses	Introduction 4
			(b) Distinguish between primary and secondary objectives	Introduction 4
Methods	Intervention	4	Define the intervention time point(s) used in the analysis	Method 2
	Participants	5	(a) List eligibility criteria and methods of selection	Method 2
			(b) Define subgroups	Method 3
			(c) Consider including a comparison group not exposed to the intervention as a secondary group of participants	Method 5 – comparison outcome
	Data sources and measurement	6	(a) List data source(s)	Method 1
			(b) Comment on data completeness, validity, and changes in data coverage over time	Method 1
	Variables	7	(a) Define all variables	Method
			_ Outcome variable(s)	5, 7
			_ Descriptive and stratifying variable(s)	5, 10
			(b) Comment on change in variable coding over time	6, 8, 9
			(c) Consider including details of variable coding in supplemental material, for example, appendix or research Web site	In protocol
	Statistical methods	8	(a) Report all statistical methods	Method
			_ Study time intervals, for example, monthly, quarterly	11 ,13 -statistical analysis
			_ Regression model, for example, ARIMA, linear, segmented	12
			B For ARIMA models, indicate the intervention function, for example, point, ramp, or step	13
			B Indicate the appropriateness of linear model(s) when applied	Discussion 10

			_ Number of preintervention, postintervention, and between intervention data points	13
			(b) Define the study period and number of preintervention data points used in forecasting	14
			(c) Indicate how autocorrelation, non-stationarity, and seasonality were tested and handled	13
			(d) Consider a lag period if intervention effects are gradual or delayed	13
			(e) Define and distinguish between primary and secondary or sensitivity analyses	16
			(f) Consider use of comparison outcome(s) and/or population(s) not exposed to the intervention(s) as secondary analyses	NA
			(g) Report statistical software used for analysis	18
Results	Participants	9	(a) Report the number of individuals and/or observations in each group analyzed	Results<br>1
			(b) Consider use of a flow diagram	NA
			(c) Describe characteristics and indicate missing data	1<br>Table 1
	Outcome data		10 (a) Report the number of outcomes examined over the study period	Table1
			(b) Report the average, minimum, and maximum number of outcomes across time intervals	Table 1
			(c) Report on data variability	2, 6, 9
			(d) Comment on outliers and ceiling or floor effects where relevant	
	Main results	11	(a) Present results using a graphical display with intervention time point(s) clearly defined	Figure 1
			(b) Consider including forecasted results graphically	Figure 1
			(c) Report absolute and/or relative change(s) and their significance, for example, clinical or policy and statistical	5, 8, 11
	Other analyses	12	Report additional results (secondary and sensitivity analyses) in the article, appendix, or research Web site	Results<br>12, 13
Discussion	Key results	13	Summarize key results with reference to study objectives	Discussion<br>1
	Context	14	(a) Provide context related to possible confounding	
			i Discuss relevant cointerventions that occurred during the study period	11
			ii Comment on the stability of participant characteristics over time	6
			iii Comment on the stability of outcome coding over time	7, 8, 9

			(b) Discuss results of comparison analyses or provide a rationale if no comparison group was considered	3, 4
	Limitations	15	(a) Discuss limitations of the study	5, 6, 7, 8, 9, 10
			(b) Comment on data variability and appropriateness of the number of data points	3
			(c) Comment on ceiling or floor effects and outliers where relevant	NA
			(d) Discuss direction and magnitude of any potential bias	7, 9, 11
	Interpretation	16	other relevant evidence	
Other information	Funding	17	List funding source(s) and role of funders	Enclosed in submission
	References	18	Reference methodological articles that support statistical methods used	Method
