# Supplementary figures and images for: Impact of a pay-for-performance scheme for long-acting reversible contraceptive (LARC) advice on contraceptive uptake and abortion in British primary care: An interrupted time series study
Source: PLoS Med. 2020 Sep 14;17(9):e1003333. doi: 10.1371/journal.pmed.1003333 (PMC7489538; doi:10.1371/journal.pmed.1003333)

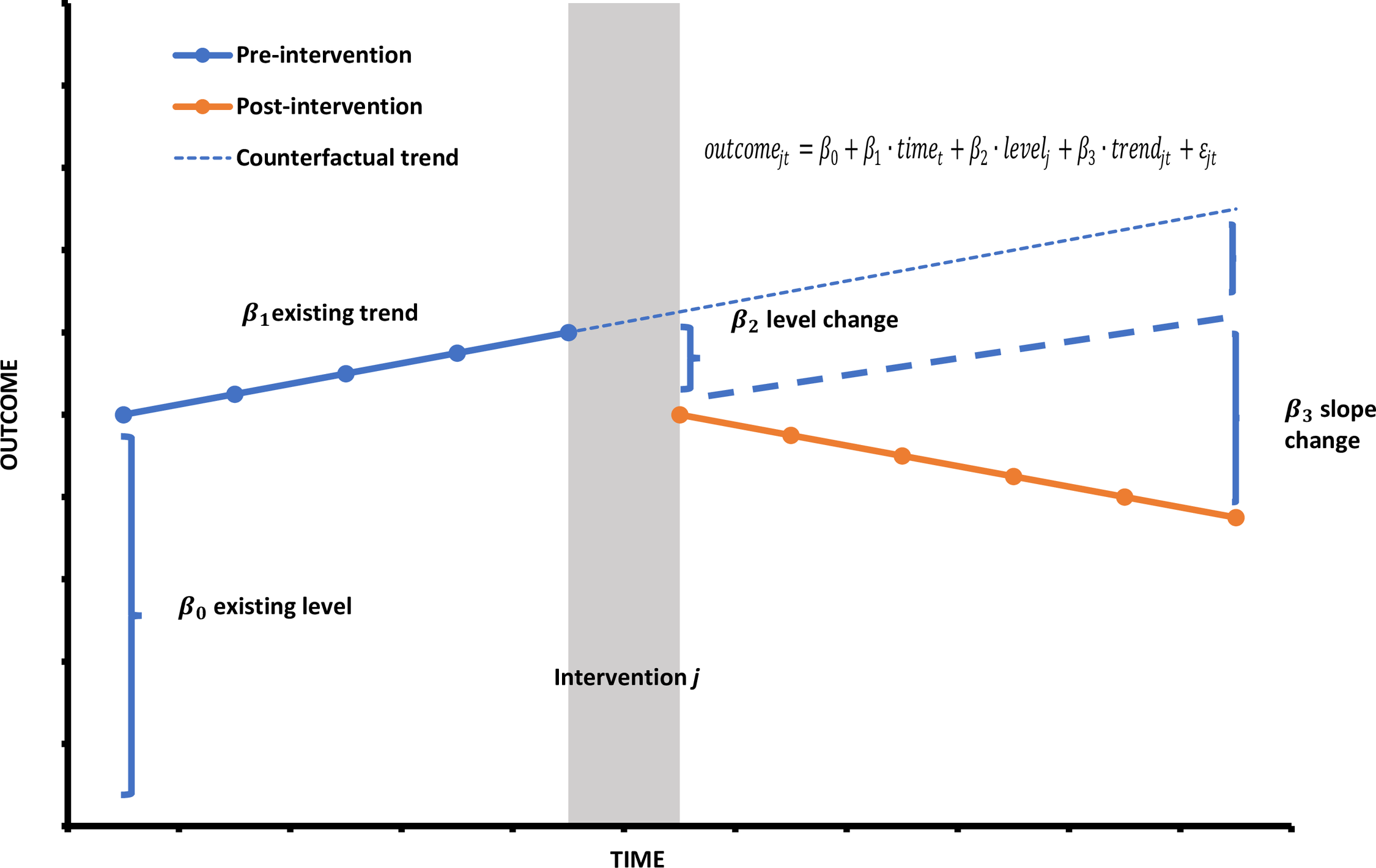

Supplement: S1 Fig — (TIF) [file pmed.1003333.s003.tif]

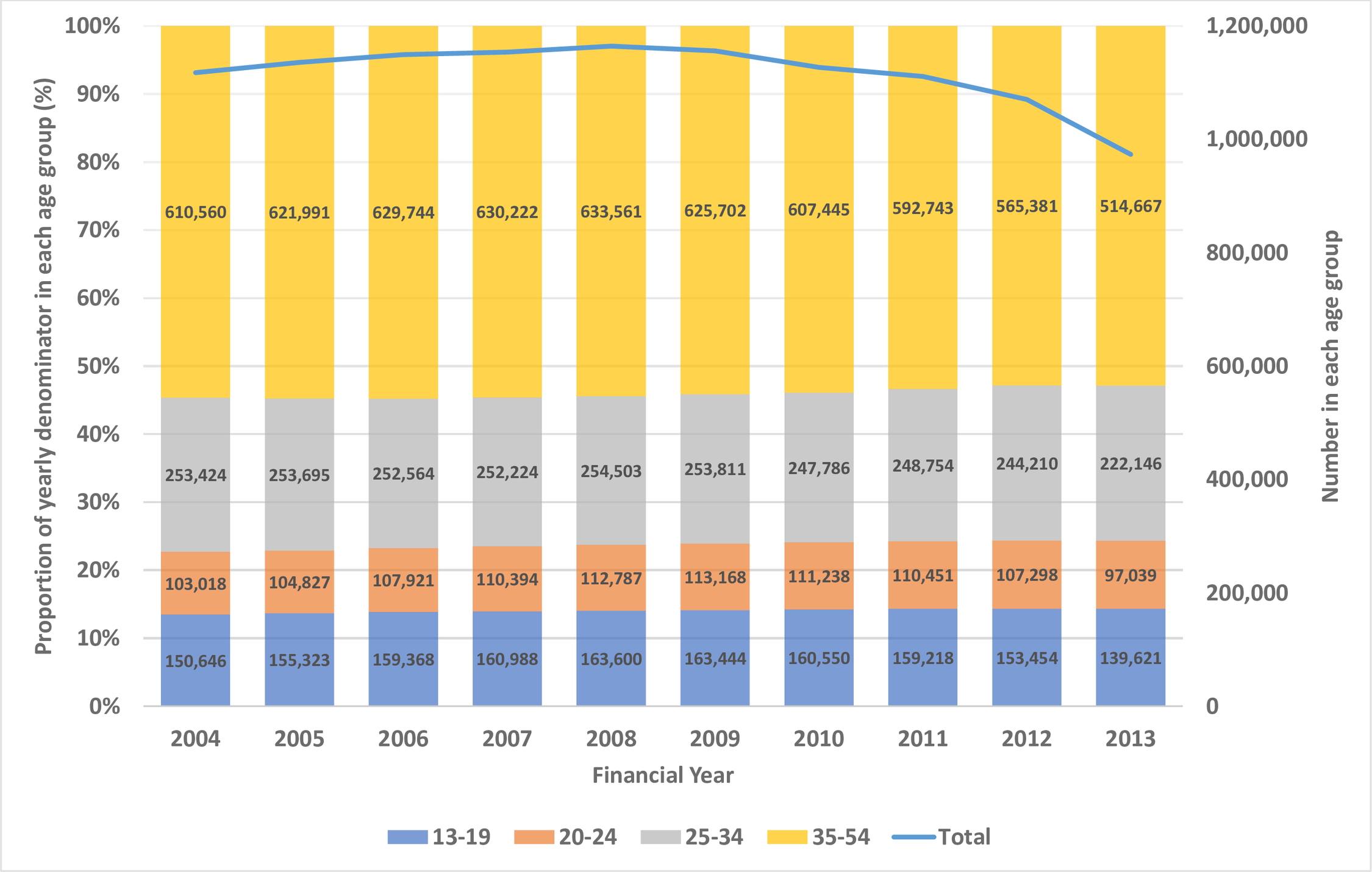

Supplement: S2 Fig — (TIF) [file pmed.1003333.s004.tif]

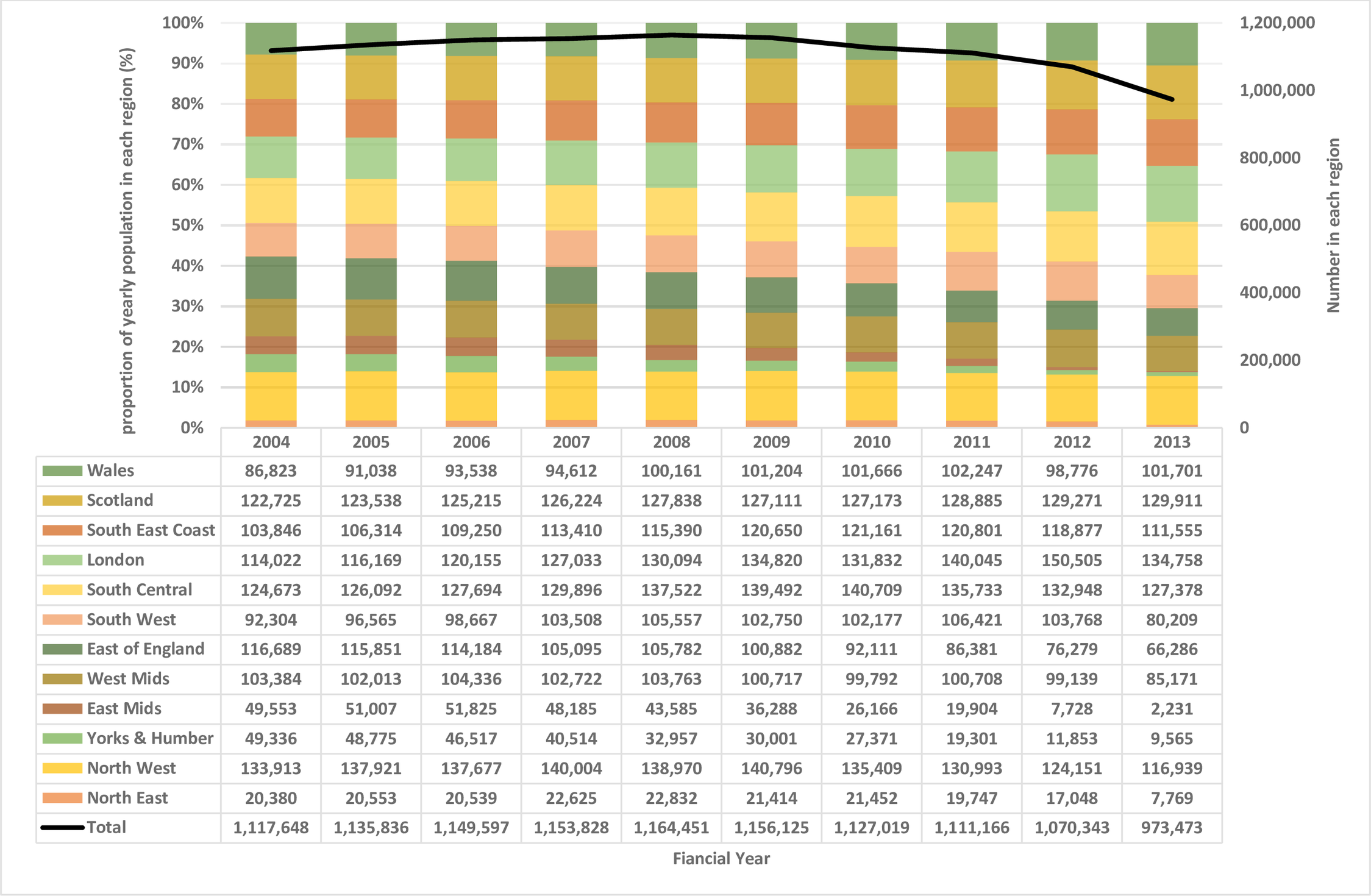

Supplement: S3 Fig — (TIF) [file pmed.1003333.s005.tif]

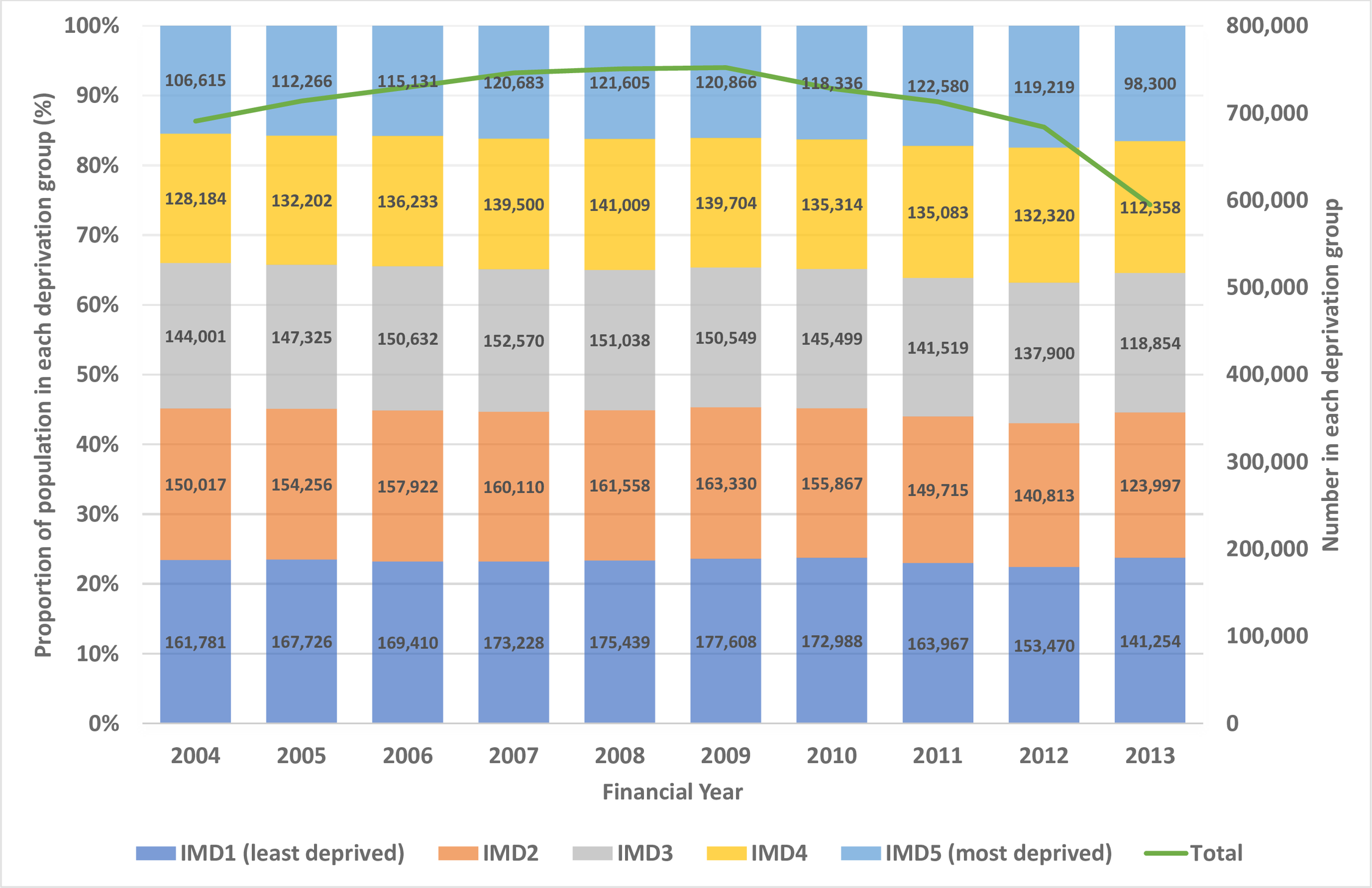

Supplement: S4 Fig — (TIF) [file pmed.1003333.s006.tif]

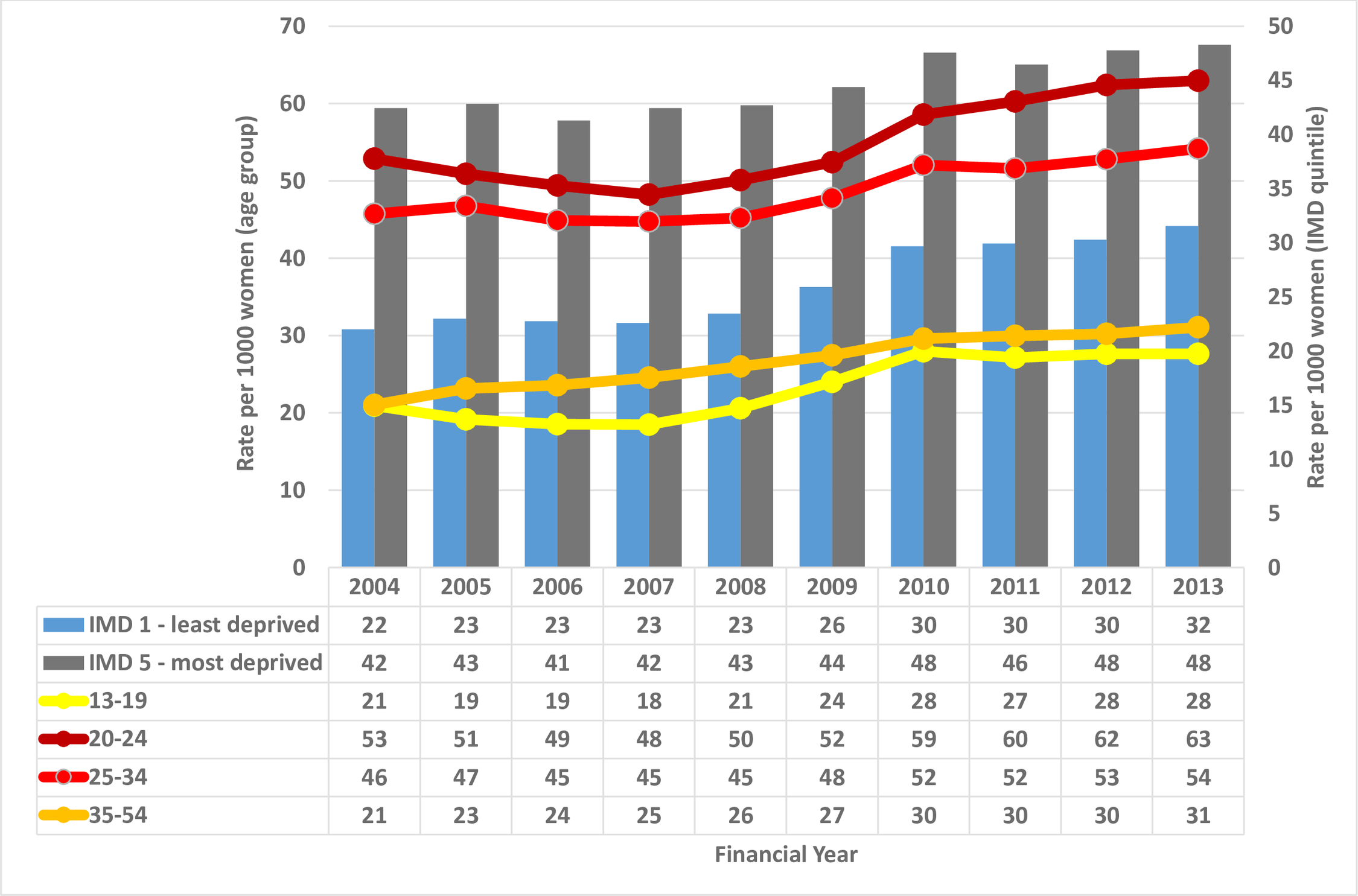

Supplement: S5 Fig — LARC, long-acting reversible contraception. (TIF) [file pmed.1003333.s007.tif]

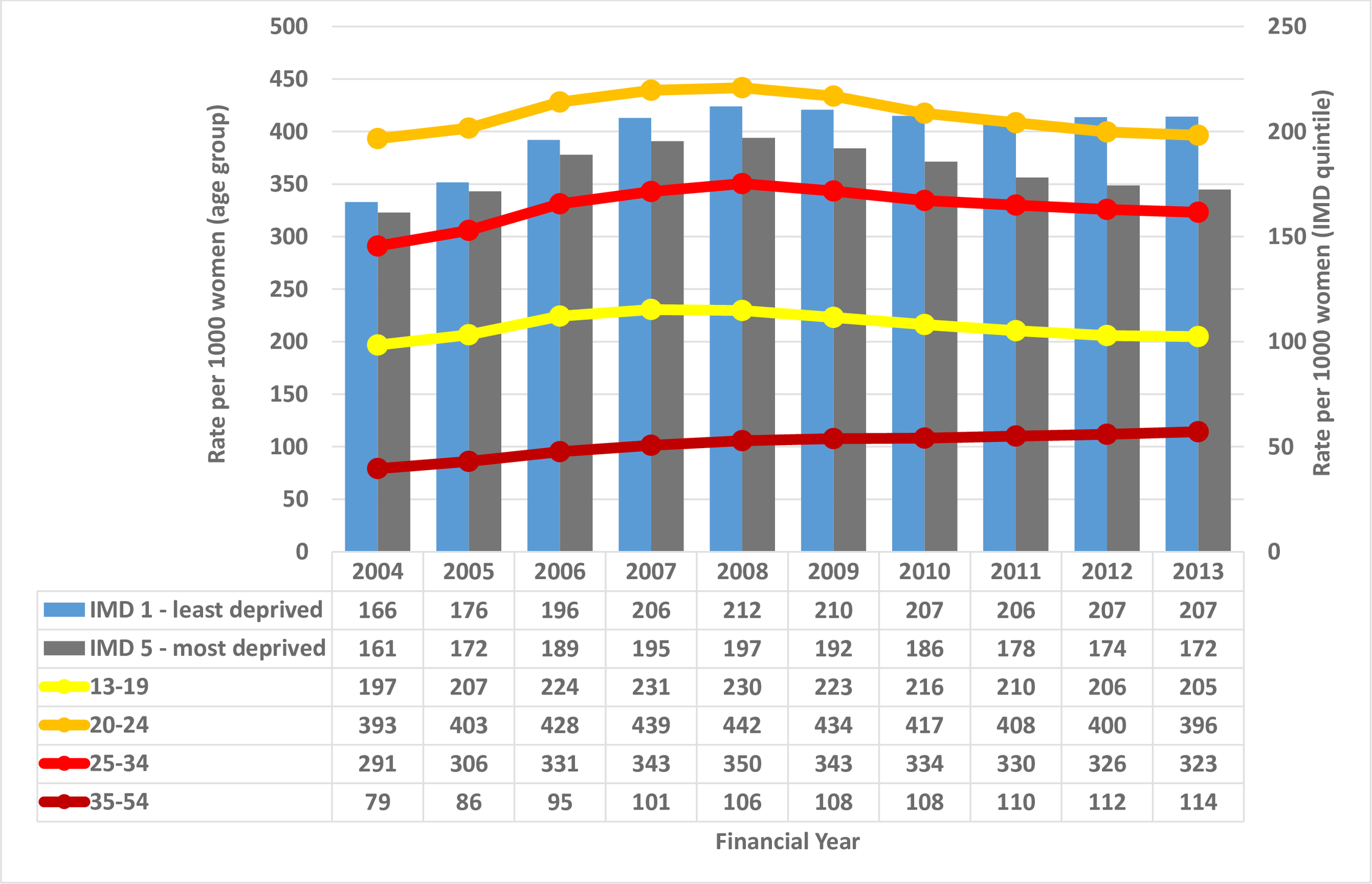

Supplement: S6 Fig — NLCH, non-LARC hormonal contraception. (TIF) [file pmed.1003333.s008.tif]

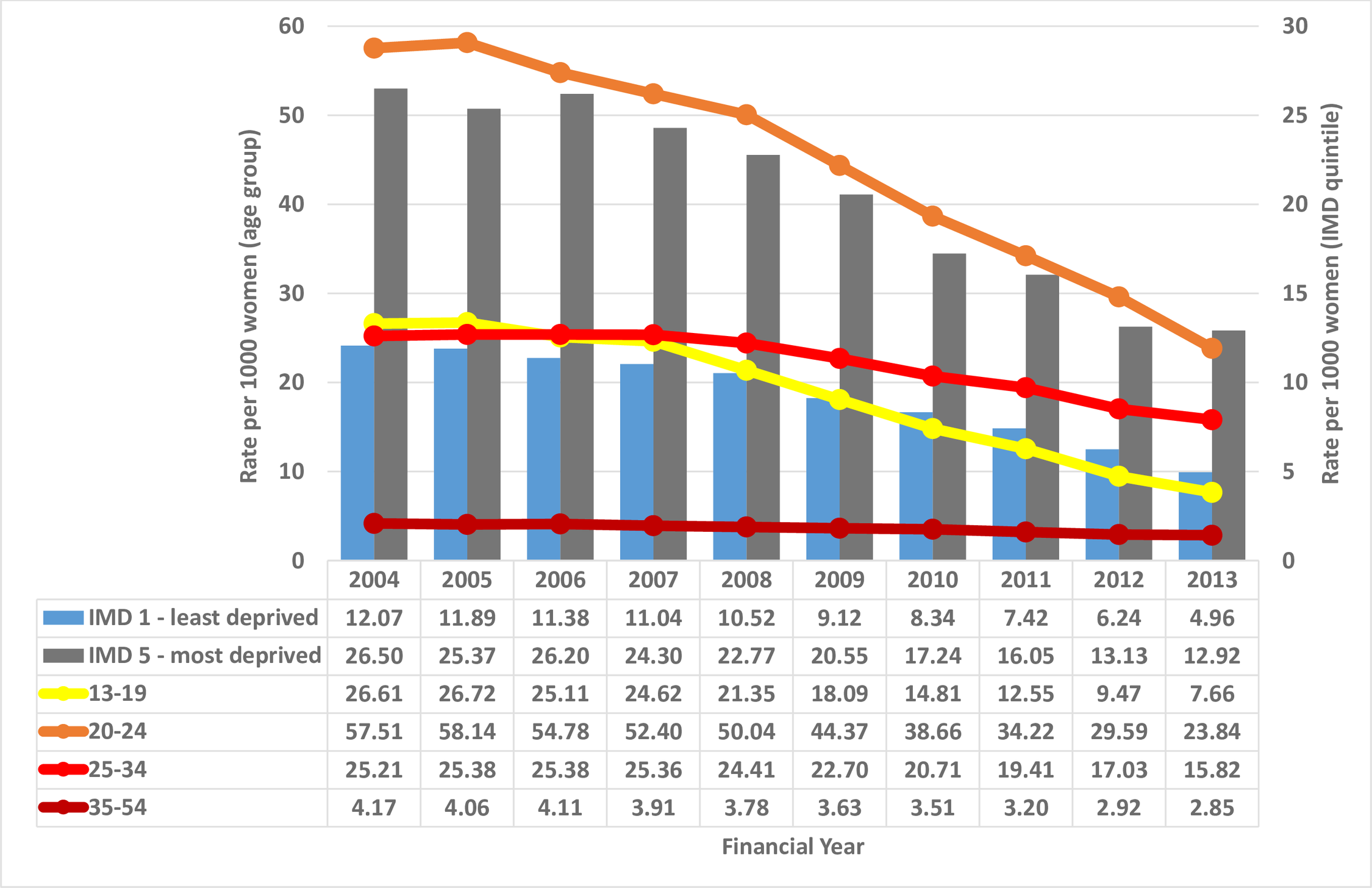

Supplement: S7 Fig — (TIF) [file pmed.1003333.s009.tif]

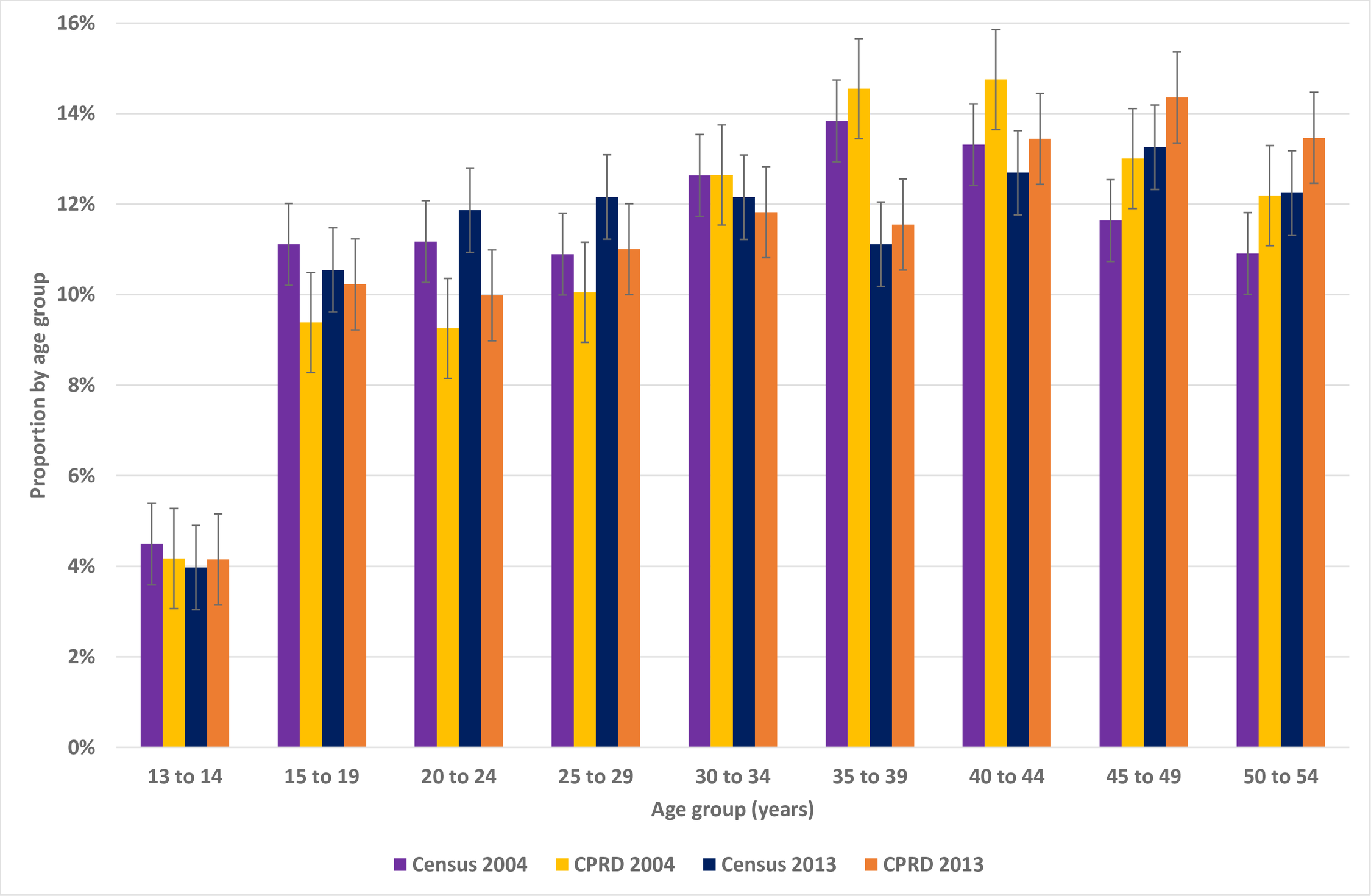

Supplement: S8 Fig — CPRD, Clinical Practice Research Datalink. (TIF) [file pmed.1003333.s010.tif]
