# Supplementary material for: Impact of a pay-for-performance scheme for long-acting reversible contraceptive (LARC) advice on contraceptive uptake and abortion in British primary care: An interrupted time series study
Source: PLoS Med. 2020 Sep 14;17(9):e1003333. doi: 10.1371/journal.pmed.1003333 (PMC7489538; doi:10.1371/journal.pmed.1003333)
Supplement: S1 Table — (A) LARC advice given as a percentage of eligible women aged 13 to 54 using an NLHC. (B) Uptake per 1,000 women aged 13 to 54 years by method of contraception 2004/2005 to 2013/2014. (C) Sensitivity analyses of outcomes with and without phase-in period. (D) Sensitivity analyses using scenarios of additional abortions. LARC, long-acting reversible contraception; NLHC, non-LARC hormonal contraception. (PDF) [file pmed.1003333.s011.pdf]

Supplementary S1 Tables

S1 A Table: LARC advice given as a percentage of eligible women aged 13 to 54 using a non-LARC hormonal contraception

Year	2004	2005	2006	2007	2008	2009	2010	2011	2012	2013
Percent	43.4	49.5	56.8	66.6	78.3	95.3	97.6	97.9	97.7	96.5

S1 B Table: Uptake per 1,000 women aged 13 to 54 years by method of contraception 2004/05 to 2013/14

Contraceptive uptake	2004	2005	2006	2007	2008	2009	2010	2011	2012	2013
POP*	29.6	34.9	40.5	44.9	49.0	52.9	56.7	61.3	65.0	69.1
CHC*	142	146	156	160	160	155	147	142	138	134
Injection	22.2	21.6	20.3	19.5	19.2	19.3	20.1	20.6	20.7	21.0
Implant	0.85	1.35	2.00	2.76	3.70	5.19	6.71	6.65	7.47	7.84
IUD*	2.13	2.14	2.02	1.95	2.11	1.94	2.16	2.10	2.02	1.95
IUS*	4.42	5.32	5.62	6.20	6.77	7.47	8.22	8.11	8.03	8.26

**POP: Progestogen-Only Pill; CHC: Combined Hormonal Contraception; IUD: Intra-Uterine Device; IUS: Intra-Uterine System*

S1 C Table: Sensitivity analysis of outcomes with and without phase-in period

Outcome (per 1,000 women)	*Pre-P4P trend*	*Change in level*	*Post-P4P trend*	*Absolute 4 years after P4P*	*Relative change 4 years after P4P*
LARC all ages- with phase-in	0.56 (0.54 to 0.59)	5.36 (5.26 to 5.45)	-0.35 (-0.42 to -0.28)	4.53	13.4%
LARC all ages - no phase-in	0.24 (0.20 to 0.28)	3.34 (3.17 to 3.52)	0.84 (0.81 to 0.86)	7.54	23.6%
NLHC all ages- with phase-in	9.25 (8.98 to 9.52)	-22.8 (-24.5 to -21.2)	-7.10 (-7.88 to -6.33)	-42.0	-16.9%
NLHC all ages - no phase-in	9.90 (9.65 to 10.2)	-8.01 (-9.09 to -6.92)	-10.4 (-10.6 to -10.2)	-60.0	-22.8%
Abortion all ages- with phase-in	-0.41 (-0.55 to -0.27)*	-2.28 (-2.99 to -1.57)*	-0.88 (-1.12 to -0.63)	-5.31	-38.3%
Abortion all ages- no phase-in	-0.41 (-0.45 to -0.38)	-1.49 (-1.62 to -1.35)	-0.85 (-0.86 to -0.83)	-4.87	-36.1%

*All values p<0.001 unless stated. *p=0.002*

S1 D Table: Sensitivity analysis of additional abortions

Abortion rate (per 1,000 women)	*Pre-P4P trend*	*Change in level*	*Post-P4P trend*	*Absolute 4 years after P4P*	*Relative change 4 years after P4P*
Additional 20%	-0.41 (-0.55 to -0.26)[1]	0.21 (-0.52 to 0.93)[2]	-1.13 (-1.38 to -0.88)	-3.60	-25.9%
Additional 40%	-0.41 (-0.55 to -0.26)[3]	2.69 (1.95 to 3.43)	-1.39 (-1.65 to -1.13)	-1.88	-13.6%
Additional 50%	-0.41 (-0.55 to -0.26)[4]	3.93 (3.18 to 4.68)	-1.52 (-1.78 to -1.26)	-1.03	-7.39%

All values p<0.001 unless stated. [1]p=0.003, [2]p=0.599, [3]p=0.003, [4]p=0.003
